# FBXO22 Suppresses Oxidative Stress-Induced ASK1 Activation and Cell Death via Ubiquitination-Dependent Degradation of TRIM48

**DOI:** 10.3390/ijms26199472

**Published:** 2025-09-27

**Authors:** Naoki Kashiwabara, Keita Nagaoka, Kenshin Nakajima, Hiroki Tsukamoto, Yoshihisa Tomioka, Isao Naguro, Hidenori Ichijo, Takuya Noguchi, Yusuke Hirata, Atsushi Matsuzawa

**Affiliations:** 1 Laboratory of Health Chemistry, Graduate School of Pharmaceutical Sciences, Tohoku University, Sendai 980-8578, Japan; naoki.kashiwabara.p7@dc.tohoku.ac.jp (N.K.); keita.nagaoka.0804@gmail.com (K.N.); kenshin1163@gmail.com (K.N.); noguctak@iwate-med.ac.jp (T.N.); 2 Department of Pharmaceutical Sciences, School of Pharmacy at Fukuoka, International University of Health and Welfare, Okawa 831-8501, Japan; tsukamoh@ihwg.jp; 3 Laboratory of Oncology, Pharmacy Practice and Sciences, Graduate School of Pharmaceutical Sciences, Tohoku University, Sendai 980-8578, Japan; yoshihisa.tomioka.a6@tohoku.ac.jp; 4 Laboratory of Bioresponse Signaling, Faculty of Pharmacy, Juntendo University, Urayasu 279-0013, Japan; i.naguro.qu@juntendo.ac.jp; 5 Laboratory of Cell Signaling, Graduate School of Pharmaceutical Sciences, University of Tokyo, Bunkyo-ku, Tokyo 113-0033, Japan; ichijo@g.ecc.u-tokyo.ac.jp; 6 Cell Signaling and Stress Responses Laboratory, Advanced Research Institute, Institute of Science Tokyo, Chiyoda-ku, Tokyo 101-0062, Japan; 7 Department of Medical Biochemistry, School of Pharmacy, Iwate Medical University, 1-1-1 Idaidori, Yahaba-cho, Shiwa-gun 028-3694, Japan

**Keywords:** FBXO22, TRIM48, ASK1, ubiquitin, protein degradation, oxidative stress

## Abstract

TRIM48 is a human-specific tripartite motif (TRIM) family protein with E3 ubiquitin ligase activity that plays a significant role in the oxidative stress response and tumor suppression. However, the mechanisms regulating TRIM48 expression remain unknown. In this study, we demonstrate that TRIM48 is targeted for ubiquitination-dependent degradation by S-phase kinase-associated protein 1 (Skp1)-Cullin1 (Cul1)-F-box protein (SCF) ubiquitin ligase complex, containing F-box protein 22 (FBXO22) as a substrate recognition subunit. We found that TRIM48 is a rapid turnover protein, as evidenced by the fast and drastic decrease in its protein expression level in the presence of a protein synthesis inhibitor cycloheximide, which was suppressed by knocking down either Skp1, Cul1 or FBXO22. Exogenous FBXO22 expression promoted K48-linked polyubiquitination and degradation of TRIM48. FBXO22 deficiency accelerated oxidative stress-induced activation of apoptosis signal-regulating kinase 1 (ASK1) and cell death, which was reversed by additional TRIM48 knockdown. Collectively, our findings identify the FBXO22 SCF complex as a key negative regulator of TRIM48-driven ASK1-activation and cell death under oxidative stress. The dysregulation of this axis may underlie human-specific pathologies, such as tumorigenesis and oxidative stress-associated disorders, highlighting its potential as a target for novel therapeutic interventions.

## 1. Introduction

Ubiquitin modification is a widely conserved post-translational modification in eukaryotes [1]. The tripartite motif (TRIM) family is the largest family of E3 ubiquitin ligases, typically possessing three conserved domains/motifs: RING-finger motif, B-box domain, and coiled-coil domain [2]. TRIM family proteins play a crucial role in diverse cellular processes, including cell death and inflammatory responses, by determining substrate specificity and the ubiquitin binding mode [2]. The number of TRIM genes varies significantly across species, ranging from 5 to 10 in lower invertebrates to 60–70 in mammals and reaching nearly 100 in humans [3,4]. Notably, a previous report identified several TRIM genes specific to primates/humans, such as TRIM43, TRIM48, TRIM49, and TRIM64, highlighting the rapid evolution of TRIM genes in primates/humans and their potential role in species-specific biological functions, such as immune adaptations [3]. These primate/human-specific TRIM genes likely arose through segmental duplication, possibly enabling primates to acquire new antiviral genes and adapt to evolving pathogens [3,4]. Although several primate/human-specific TRIM family proteins, such as TRIM43 [5] and TRIM64 [6], have been shown to regulate antiviral responses and pro-inflammatory signaling, respectively; most of their functional roles remain uncharacterized.

TRIM48 is a human-specific TRIM family protein, containing the RING-finger motif and B-box domain, while lacking a coiled-coil domain [3,4]. Previously, we demonstrated that TRIM48 facilitates the activation of an oxidative stress-responsive mitogen-activated protein kinase kinase kinase (MAP3K), apoptosis signal-regulating kinase 1 (ASK1), via the ubiquitination and proteasomal degradation of its negative regulator protein arginine methyltransferase 1 (PRMT1) [7]. In response to oxidative stress, ASK1 is auto-phosphorylated to activate downstream stress-responsive MAP kinases, including c-Jun N-terminal kinase (JNK) and p38, thereby inducing multiple cellular responses such as cell death and inflammation, depending on the situation and context [8]. ASK1 activation is meticulously regulated by diverse interacting molecules that form a huge complex, called ASK1 signalosome (~1500–2000 kDa) [9], through various post-translational modifications, such as phosphorylation, ubiquitination, and methylation. The role of ASK1 in human health is significant, as its dysregulation has been associated with a wide range of diseases, from cancers to neurodegenerative disorders; consequently, there is a growing interest in developing ASK1 modulators as potential therapeutics [8]. Although multiple studies have demonstrated the crucial role of TRIM48 in tumor suppression [7,10,11], the regulatory mechanism of TRIM48 activity and expression remains unknown.

Herein, we report that TRIM48 is specifically ubiquitinated by the S-phase kinase-associated protein 1 (Skp1)-Cullin1 (Cul1)-F-box protein (SCF) ubiquitin ligase complex [12], containing F-box protein 22 (FBXO22) as a substrate recognition subunit, targeting it for proteasomal degradation. FBXO22 knockdown attenuated H_2_O_2_-induced ASK1 phosphorylation and cell death, and additional TRIM48 knockdown reversed these effects, indicating a suppressive role of FBXO22 in ASK1 activation and cell death under oxidative stress. These findings offer insights into the human-specific mechanism by which TRIM48 regulates ASK1 activation and cell death during oxidative stress, shedding light on a potentially significant aspect of human biology and pathology, including cancer.

## 2. Results

### 2.1. TRIM48 Protein Undergoes Proteasomal Degradation

**Figure 1:**
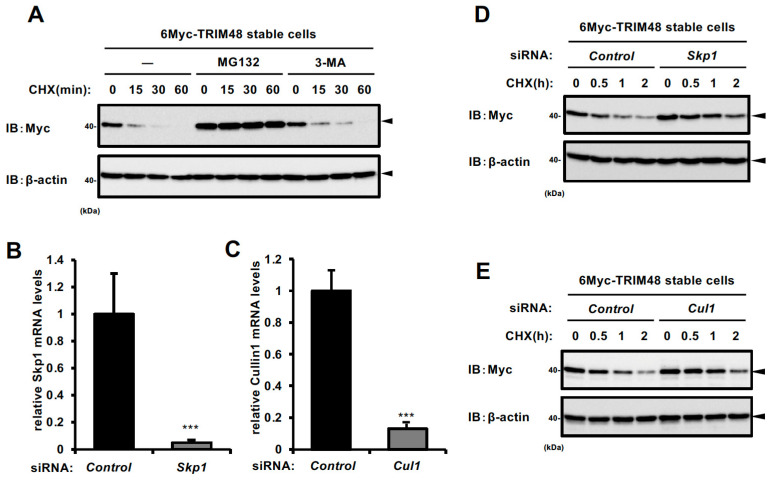
TRIM48 protein undergoes proteasomal degradation. (**A**) HEK293A cells stably expressing 6Myc-TRIM48 were treated with cycloheximide (CHX) along with 5 µM MG132 or 5 mM 3-MA for the indicated times. Cell lysates were immunoblotted with the indicated antibodies. (**B**,**C**) Relative mRNA levels of Skp1 or Cul1 in HEK293A cells stably expressing 6Myc-TRIM48 were transfected with the indicated siRNAs for 48 h. Data shown are the mean  ±  SD (*n*  =  3). (**D**,**E**) HEK293A cells stably expressing 6Myc-TRIM48 were transfected with the indicated siRNA for 48 h, and treated with CHX for 0, 0.5, 1, or 2 h. Cell lysates were immunoblotted with the indicated antibodies. *** *p* < 0.001.

We previously reported that under oxidative stress, TRIM48 interacts with PRMT1, a negative regulator of ASK1, and promotes PRMT1 ubiquitination and degradation, thereby enhancing ASK1 activation and cell death [7]. In this report, we observed that upon cycloheximide (CHX) treatment, TRIM48 exhibited significantly faster degradation kinetics than PRMT1, with a half-life of less than 4 h compared to approximately 12 h for PRMT1, which led to an assumption that TRIM48 expression might be dynamically regulated at the protein level. We first investigated the degradation mechanism of TRIM48 to address this possibility. To this end, we generated HEK293A cells stably expressing 6Myc-TRIM48 and treated them with a protein synthesis inhibitor CHX, with MG132, a proteasome inhibitor, or 3-methyladenine (3-MA), an autophagy inhibitor that targets phosphatidylinositol 3-kinase (PI3K), thereby inhibiting autophagy upstream of microtubule-associated protein 1 light chain 3-II (LC3-II) formation [13]. As shown in Figure 1A, TRIM48 expression was rapidly reduced upon CHX treatment, with a half-life of ~15 min, which was apparently inhibited with MG132 treatment but not 3-MA, suggesting that TRIM48 is a rapid turnover protein constitutively degraded by the proteasome. It is worth noting that MG132 treatment led to the accumulation of K48-linked ubiquitin under the same experimental conditions, whereas 3-MA treatment suppressed the bafilomycin A1 (an autophagosome-lysosome fusion inhibitor)-induced accumulation of LC3-II, confirming that proteasome inhibition and autophagy inhibition were effectively achieved under these conditions (Figure S1A,B). Next, we aimed to identify the E3 ubiquitin ligase responsible for TRIM48 ubiquitination and degradation. In our previous study, we performed a mass spectrometry analysis to identify proteins interacting with TRIM48 expressed in HEK293A cells, and identified Skp1, a component of the SCF complex, as a binding molecule, as well as several proteasomal subunits and PRMT1 [7]. Therefore, we focused on the SCF complex as a potential E3 ligase for TRIM48 ubiquitination. The SCF complex is a multi-subunit complex composed of Skp1, Cul1, and an F-box protein [12]. The F-box protein determines the substrate specificity of the SCF complex, and SCF-mediated ubiquitination plays a crucial role in various biological processes, including cell proliferation, differentiation, and cell death [12]. To examine the involvement of the SCF complex in TRIM48 degradation, we knocked down Skp1 and Cul1 in HEK293A cells (Figure 1B,C), and treated cells with CHX to assess TRIM48 degradation. As shown in Figure 1D,E, the knockdown of either Skp1 or Cul1 inhibited TRIM48 degradation upon CHX treatment. These results suggest that TRIM48 is targeted for ubiquitination and proteasomal degradation by the SCF complex.

### 2.2. FBXO22 Serves as a Recognition Subunit for TRIM48 Within the SCF Complex

**Figure 2:**
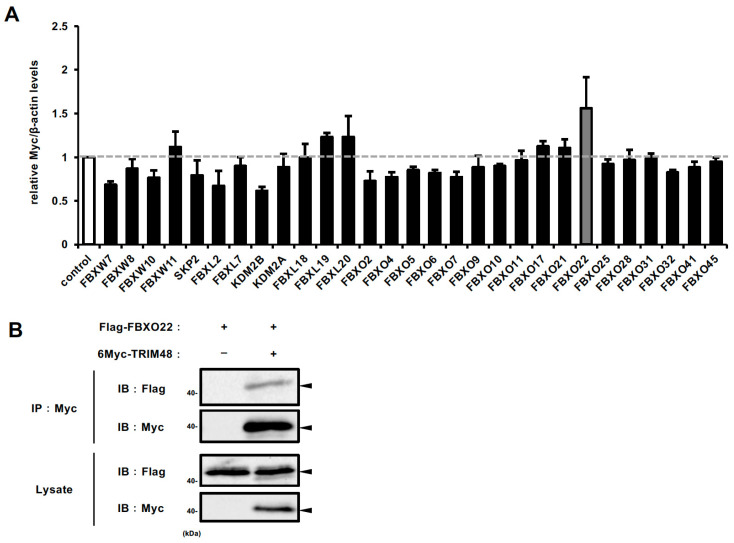
FBXO22 serves as a recognition subunit for TRIM48 within the SCF complex. (**A**) 6Myc-TRIM48 stably expressing HEK293A cells were transfected with 29 siRNAs targeting the indicated genes encoding F-box proteins for 48 h, and cell lysates were immunoblotted with anti-Myc and anti-β-actin antibodies. The graph shows the average value of the Myc band intensity with that of β-actin (mean ± SD, *n* = 3). The value of the control siRNA transfection was set as 1. (**B**) HEK293A cells were transfected with the indicated constructs for 24 h and then treated with 5 μM MG132 for 6 h. The cell lysates were immunoprecipitated with an anti-Myc antibody and subjected to immunoblotting with the indicated antibodies.

We then sought to identify the specific F-box protein responsible for TRIM48 degradation. From nearly 70 F-box proteins [14], we selected 29 candidates reported to be upregulated in at least one cancer type based on the Oncomine database [15], and implicated in cancer development and progression in previous reports. HEK293A cells stably expressing 6Myc-TRIM48 were transfected with siRNAs, and cell lysates were subjected to immunoblot analysis. Among the 29 candidates, F-box protein 22 (FBXO22) knockdown resulted in the most pronounced increase in TRIM48 protein levels (Figure 2A and Figure S2). Furthermore, a co-immunoprecipitation experiment using anti-Flag antibody demonstrated an interaction between 6Myc-TRIM48 and Flag-FBXO22 in HEK293A cells (Figure 2B). These collectively suggest that FBXO22 is an F-box protein that recognizes TRIM48 as a substrate of the SCF complex.

### 2.3. SCF FBXO22 Promotes TRIM48 Ubiquitination and Degradation

**Figure 3:**
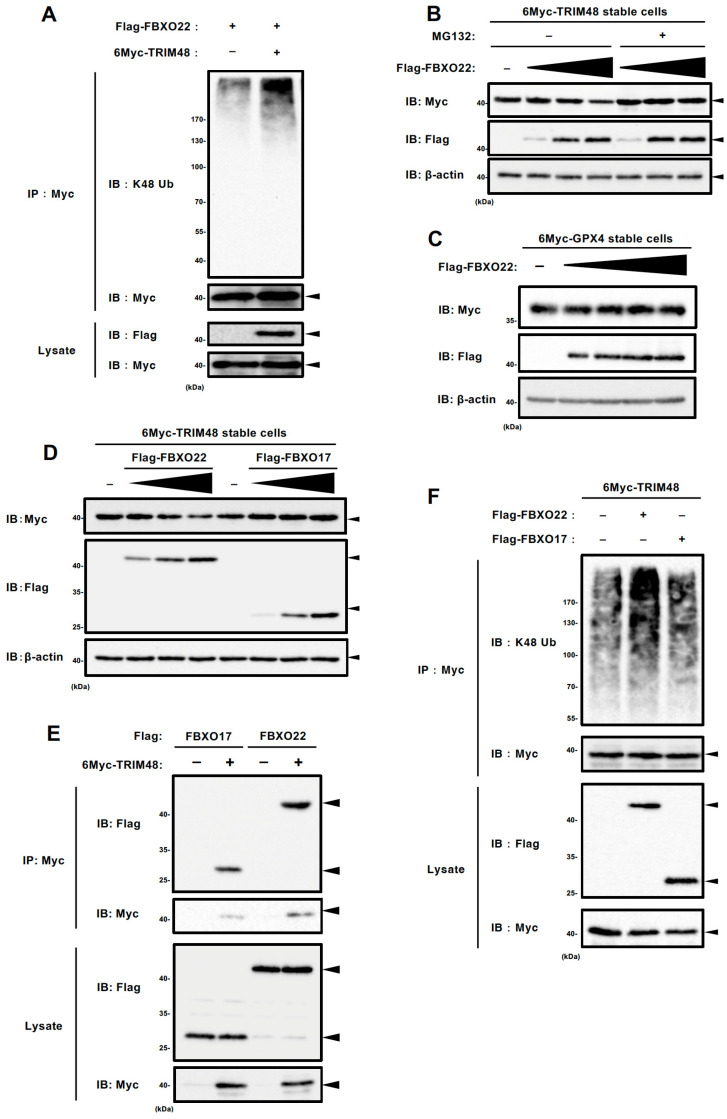
SCF FBXO22 promotes TRIM48 ubiquitination and degradation. (**A**) HEK293A cells were transfected with the indicated plasmids, and the cell lysates were subjected to an in vivo ubiquitination assay, followed by immunoblotting with the indicated antibodies. (**B**) HEK293A cells stably expressing 6Myc-TRIM48 were transfected with increasing concentrations of Flag-FBXO22 plasmid, treated with DMSO (MG132 solvent) or 5 µM MG132 for 6 h, and the cell lysates were immunoblotted with the indicated antibodies. (**C**) HEK293A cells stably expressing 6Myc-GPX4 were transfected with increasing the concentration of Flag-FBXO22 plasmid, and the cell lysates were immunoblotted with the indicated antibodies. (**D**) HEK293A cells stably expressing 6Myc-TRIM48 were transfected with increasing concentrations of the indicated plasmids for 24 h. Cell lysates were immunoblotted with the indicated antibodies. (**E**) HEK293A cells were transfected with the indicated plasmids and treated with 5 μM MG132 6 h before collection. The cell lysates were immunoprecipitated with an anti-Myc antibody and subjected to immunoblotting with the indicated antibodies. (**F**) HEK293A cells were transfected with the indicated plasmids, and the cell lysates were subjected to an in vivo ubiquitination assay, followed by immunoblotting with the indicated antibodies.

To investigate whether the FBXO22 SCF complex, hereafter called SCF FBXO22, involves ubiquitination and degradation of TRIM48, we first performed an in vivo ubiquitination assay. We observed that K48-linked polyubiquitination of 6Myc-TRIM48 was clearly increased when co-expressed with Flag-Fbxo22 in HEK293A cells (Figure 3A and Figure S3A). In accordance with this, when 6Myc-TRIM48 stable cells were transfected with Flag-FBXO22 expression plasmid, TRIM48 expression decreased in a plasmid dose-dependent manner, which was partially inhibited by the presence of MG132, a proteasome inhibitor (Figure 3B and Figure S3B). In addition, Flag-FBXO22 overexpression in 6Myc-glutathione peroxidase 4 (GPX4) stable HEK293A cells did not alter 6Myc-GPX4 expression, confirming that FBXO22 targets TRIM48, instead of the 6Myc tag, leading to ubiquitination-dependent degradation (Figure 3C and Figure S3C). We examined the effect of overexpressing another F-box protein, FBXO17, to assess the specificity of TRIM48 degradation. As expected, TRIM48 was efficiently degraded upon FBXO22 overexpression, whereas FBXO17 failed to induce any degradation, underscoring the unique role of FBXO22 in this process (Figure 3D and Figure S3D). Altogether, these results suggest that TRIM48 is specifically ubiquitinated and degraded by SCF FBXO22. We performed co-immunoprecipitation of Flag-FBXO17 and 6Myc-TRIM48 to demonstrate the binding specificity. Surprisingly, Flag-FBXO17 co-immunoprecipitated with 6Myc-TRIM48 to a similar extent as Flag-FBXO22 (Figure 3E). On the other hand, Flag-FBXO17 expression did not elevate the K48-linked ubiquitination level of TRIM48, in contrast to Flag-FBXO22 (Figure 3F and Figure S3E). These results suggest that, while FBXO17 can interact with TRIM48 similarly to FBXO22, only FBXO22 specifically facilitates its ubiquitination and degradation.

### 2.4. SCF FBXO22 Protects Against Oxidative Stress-Induced Cell Death by Counteracting TRIM48

**Figure 4:**
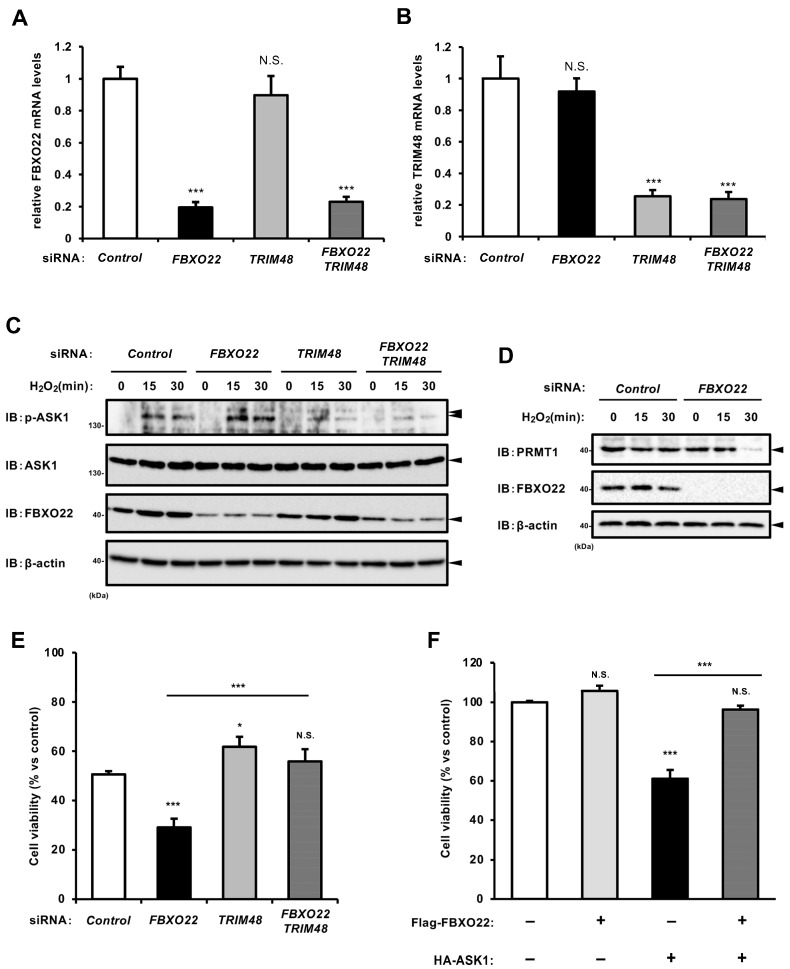
SCF FBXO22 protects against oxidative stress-induced cell death by counteracting TRIM48. (**A**,**B**) Relative mRNA levels of FBXO22 (**A**) and TRIM48 (**B**) in HEK293A cells transfected with the indicated siRNAs for 48 h. (**C**,**D**) HEK293A cells were transfected with the indicated siRNAs for 48 h, and treated with 0.5 mM H_2_O_2_ for the indicated times. Cell lysates were subjected to immunoblotting with the indicated antibodies. (**E**) Cell viability of HEK293A cells transfected with the indicated siRNAs for 48 h, followed by treatment with 0.5 mM H_2_O_2_ for 12 h. (**F**) Cell viability of HEK293A cells transfected with the indicated plasmids for 24 h. The data shown are the mean ± SD (*n* = 3). * *p* < 0.05; *** *p* < 0.001; N.S., not significant.

We showed that SCF FBXO22 facilitates the ubiquitination-dependent degradation of TRIM48. To explore the physiological significance of FBXO22-mediated regulation of TRIM48 expression, we examined whether FBXO22 knockdown affects oxidative stress-induced ASK1 activation and cell death in a TRIM48-dependent manner. As shown in Figure 4A–C, FBXO22 knockdown enhanced H_2_O_2_-induced ASK1 phosphorylation, whereas TRIM48 knockdown strongly suppressed it, regardless of FBXO22 knockdown. We observed that PRMT1 expression levels were consistently reduced upon H_2_O_2_ treatment in FBXO22 knockdown cells (Figure 4D). In line with this result, FBXO22 knockdown significantly reduced cell viability upon H_2_O_2_ treatment, while additional TRIM48 knockdown effectively reversed it (Figure 4E). In addition, the co-expression of Flag-FBXO22 prevented reduced cell survival caused by HA-ASK1 overexpression, which likely induced apoptosis (Figure 4F). Taken together, these data suggest that SCF FBXO22 negatively regulates ASK1 activation under oxidative stress by targeting TRIM48 for ubiquitination and proteasomal degradation, thereby suppressing oxidative stress-induced cell death.

## 3. Discussion

**Figure 5:**
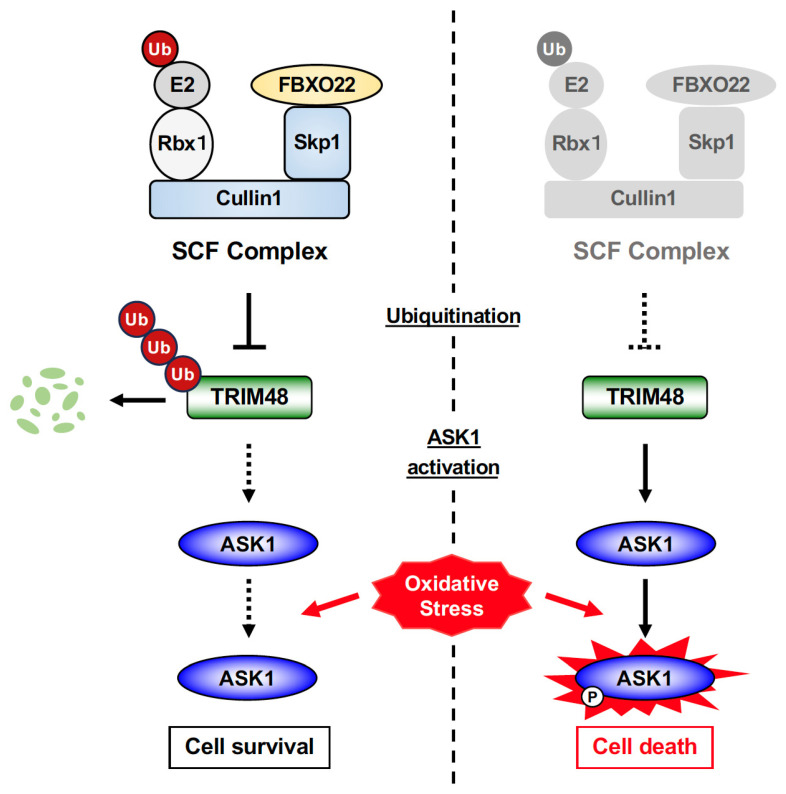
The proposed model. SCF FBXO22 facilitates ubiquitination and degradation of TRIM48, thereby inhibiting ASK1 activation and protecting cells from oxidative stress-induced death to maintain cell survival. In the absence of SCF FBXO22, TRIM48 protein levels remain elevated, promoting ASK1 activation and cell death under oxidative stress [7]. Ub: ubiquitin, P: phosphorylation, Rbx1: RING-box protein 1.

In this study, we demonstrated that FBXO22 SCF promotes TRIM48 ubiquitination and degradation, thereby suppressing oxidative stress-induced ASK1 activation and cell death (Figure 5). To the best of our knowledge, this report provides the first evidence of a regulatory mechanism controlling TRIM48 expression, which may shed light on the pathophysiological significance of TRIM48, particularly in cancers. A previous study revealed that TRIM48 is downregulated in multiple human glioblastoma cell lines, and forced expression of TRIM48 suppressed their growth [16]. A recent paper showed that thymidine kinase 1 directly binds to PRMT1 and disrupts its association with TRIM48, which prevents PRMT1 degradation and, in turn, enhances glycolysis, thus progressing HCC [10]. The interaction between TRIM48 and PRMT1 is also counteracted by Kinectin 1, which prevents PRMT1 ubiquitination and degradation, thereby promoting gastric cancer cell proliferation and a poorer prognosis [11]. Nevertheless, evidence supporting the involvement of TRIM48 in cancer development and progression remains limited, necessitating further investigation. There is also a pressing need for a highly sensitive antibody to detect the endogenous TRIM48 protein. Despite extensive efforts, including evaluating multiple commercial antibodies and generating custom antibodies using the TRIM48 small peptide or recombinant protein immunogens, we were unable to obtain or develop a suitable one. This limitation prevented us from fully investigating the importance of the FBXO22-dependent regulatory mechanism for TRIM48 expression, necessitating further efforts to develop a suitable antibody for TRIM48.

On the other hand, the role of FBXO22 in cancer development and progression has been the subject of extensive investigation in recent studies, establishing its general role in tumorigenesis and tumor progression [17]. FBXO22 has been shown to promote the ubiquitination of tumor suppressors such as p53 [18], p21 [19], KLF4 [20], LKB1 [21], and PTEN [22], thereby negatively regulating their activity, leading to cancer progressions including HCC, colorectal and breast cancers. Interestingly, FBXO22 plays a suppressive role in the migration and metastasis of certain cancer cell types, depending on the context. FBXO22 overexpression promotes cell proliferation and colony formation in breast cancers, while suppressing epithelial–mesenchymal transition (EMT) due to FBXO22-mediated ubiquitination and degradation of Snail family zinc finger 1 (SNAI1), also referred to as SNAIL, a key factor triggering EMT [23]. The oncoprotein HDM2, often overexpressed in various cancers, is targeted for ubiquitination and degradation by FBXO22 in breast cancer cells [24]. This degradation leads to decreased invasion and metastasis in breast cancer, and a negative correlation between FBXO22 and HDM2 expression has been observed in breast cancer patients [24]. BTB and CNC homology 1 (Bach1) is a transcription factor involved in various cellular processes, including cell cycle, senescence, angiogenesis, and cancer progression [17]. FBXO22-mediated degradation of Bach1 has been shown to inhibit cell migration in lung cancer cells [25]. Intriguingly, although ASK1 has been widely recognized as a tumor suppressor that promotes cancer cell death [26], emerging evidence suggests that ASK1 can play a pro-metastatic role in several types of cancers, including chondrosarcoma, lung, and ovarian cancers [27,28,29]. Given the opposing roles of FBXO22 and ASK1 in cancer as described above, it can be assumed that FBXO22 negatively regulates ASK1 activity through TRIM48 ubiquitination and degradation, thereby influencing cancer development and progression in a context-dependent manner. The precise mechanisms underlying these opposing effects require further investigation.

FBXO22 plays a crucial role in not only cancer biology but also various physiological processes. It also protects neurons from rotenone-induced neurotoxicity and Parkinson’s disease (PD) symptoms in rats, by possibly targeting the PH domain and leucine rich repeat protein phosphatase 1 (PHLPP1) for ubiquitination-dependent degradation, leading to AKT, also known as protein kinase B [30], inactivation. Considering the causal roles of ASK1 in neurodegenerative diseases, including PD30 [30], FBXO22 might alleviate neurotoxicity by suppressing ASK1 activity as well as activating the AKT pathway [30]. FBXO22 has also been implicated in neuronal plasticity, neurotransmission, and viral replication [30,31,32]. While ASK1 has been closely associated with antiviral responses, its precise role in neuronal activities remains unclear, implying that FBXO22 may modulate neuronal activity by targeting TRIM48 through mechanisms independent of ASK1. However, thus far, besides PRMT1, no other ubiquitination substrate of TRIM48 has been reported, highlighting the need for further studies to identify additional targets.

TRIM48 is a human-specific TRIM family member that may underlie species-specific aspects of cellular regulation [3,4]. Beyond cancer, notably, TRIM48 has been particularly implicated in facioscapulohumeral muscular dystrophy (FSHD) [33], which is caused by the ectopic expression of a primate-specific transcription factor double homeobox 4 (DUX4) in muscles. TRIM48 is one of its representative downstream genes, with compelling evidence reporting its elevated expression in this pathology [20,34]. Although the disease etiology is still unclear, ectopic DUX4 expression has been closely associated with oxidative stress and apoptotic cell death in muscle cells [35,36], phenotypes that mirror the cellular effects of the FBXO22–TRIM48–ASK1 signaling axis uncovered in this study. The pronounced heterogeneity in FSHD progression, together with the absence of a clear link between DUX4 expression levels and disease severity [37], raises the possibility that FBXO22 expression changes or functional impairment could underlie the TRIM48-dependent promotion of myocyte death; aberrant TRIM48 expression resulting from FBXO22 deficiency could intensify oxidative stress-induced ASK1 activation and apoptotic cell death, underscoring a potential interplay among these factors. Investigating the functional relationship between the FBXO22/TRIM48 axis and DUX4-related pathologies could yield valuable insights into the molecular underpinnings of human-specific diseases. Future studies could assess whether targeting FBXO22 or TRIM48 could modulate DUX4-driven stress responses, potentially offering avenues for therapeutic intervention in FSHD and related disorders. Overall, this study highlights the role of the human-specific protein TRIM48 in shaping conserved signaling networks and opens up new avenues in evolutionary biology and precision medicine.

## 4. Materials and Methods

### 4.1. Reagents

All reagents were obtained from commercial suppliers: Fetal Bovine Serum (FBS) (Nichirei Biosciences, Tokyo, Japan), cycloheximide (Sigma, Kanagawa, Japan), MG132 (Enzo Life Sciences, Farmingdale, NY, USA), 3-Methyladenine Autophagy inhibitor (3-MA, AdipoGen, San Diego, CA, USA), bafilomycin A1 (Santa Cruz, Dallas, TX, USA), Hydrogen Peroxide (H_2_O_2_) (Wako, Tokyo, Japan).

### 4.2. Cell Culture and Transfection

The cells used in this study were cultured in a CO_2_ incubator at 37 °C with 5% CO_2_-95% air as the gas phase. HEK293A cells were maintained and passaged in Dulbecco’s Modified Eagle Medium (DMEM) (Nacalai Tesque, Kyoto, Japan) containing 5% FBS and 1% penicillin-streptomycin solution. Plasmid transfection was performed using Polyethylenimine “Max” (PEI MAX) (Polysciences, Warrington, PA, USA), according to the manufacturer’s instructions.

### 4.3. Plasmids

Samples of cDNAs encoding human FBXO22 or FBXO17 were obtained by conducting PCR and were inserted into pcDNA3.2 with Flag tag plasmids. TRIM48 was inserted into pcDNA3 plasmid tagged with 6Myc, as described previously [7]. ASK1 was inserted into pcDNA3 plasmid tagged with HA, as described previously [7,38].

### 4.4. Immunoblot Assay

Cells were lysed in ice-cold DISC lysis buffer containing 20 mM Tris-HCl, pH 7.4, 150 mM NaCl, 1% Triton-X100, 10% Glycerol, or IP lysis buffer containing 20 mM Tris-HCl, pH 7.4, 150 mM NaCl, 10 mM EDTA-2Na pH 8.0, 1% Triton-X100, 1% Sodium deoxycholate, and 1% protease inhibitor cocktail (Nacalai Tesque); in Figure 4C, DISC lysis buffer with phosphatase inhibitor cocktail (Nacalai Tesque) was used. After centrifugation, the cell extracts were resolved by SDS-PAGE, and were analyzed as described previously. The antibodies used for immunoblotting were against ASK1, Myc (clone 9E10), β-actin (Santa Cruz), Flag (clone 1E6) (Wako), K48-linked ubiquitin (Cell Signaling, Danvers, MA, USA), PRMT1 (Abcam, Cambridge, UK), LC3 (MBL, Tokyo, Japan) and phospho-ASK1 (Thr-845) [39]. The blots were developed [7] with ECL (Merck Millipore, Burlington, MA, USA), and detected with ChemiDoc Touch Imaging System (BioRad, Hercules, CA, USA). The uncropped immunoblot data are provided in Figure S4.

### 4.5. Cell Viability Assay

HEK293A cells were seeded on 96-well plates. After H_2_O_2_ treatment, cell viability was determined using Cell Titer 96 Cell Proliferation Assay (Promega, Madison, WI, USA), according to the manufacturer’s protocol. The absorbance was read at 490 nm using a microplate reader (iMark microplate reader, Bio-Rad, Hercules, CA, USA). Data are normalized to control without stimulus, unless noted otherwise.

### 4.6. RNA Extraction from Cell Lines

RNA extraction from cells was performed using Sepasol-RNA I (Nacalai Tesque). Cells seeded and stimulated in 24-well plates were washed with PBS and then lysed by adding 200 μL of Sepasol. A total of 100 μL of chloroform/isoamylalcohol (50:1) was added to this and mixed well. After allowing to stand for 3 min, samples were centrifuged at 15,000 rpm and 4 °C for 15 min, and the upper layer was transferred to another microtube. An equal volume of 2-propanol was added, followed by thorough mixing and a 10 min incubation period. After centrifugation at 15,000 rpm and 4 °C for 10 min, the precipitate was washed with 70% ethanol, air-dried, and dissolved in nuclease-free water. RNA concentration was calculated by measuring OD260.

### 4.7. qRT-PCR Analysis

Total RNA was extracted using Sepasol RNA I Super (Nacalai Tesque) and then reverse transcribed into cDNA with High-Capacity cDNA Reverse Transcription Kit (Applied Biosystems, Waltham, MA, USA) according to the manufacturer’s instructions. Relative mRNA levels of target genes were determined by real-time quantitative fluorescence PCR (qRT-PCR) using Luna Universal qPCR Master Mix (New England Biolabs, Ipswich, MA, USA) according to the manufacturer’s protocol, and normalized with those of GAPDH. 

Sequences of primers used are as follows:

Skp1-forward, 5′-CACCCACCACAAGGATGACC-3′;

Skp1-reverse, 5′-TGGGCTTCCTCCTCTTCAGT-3′;

Cullin1-forward, 5′-CTCAACAATGGGAAGATTATCG-3′;

Cullin1-reverse, 5′-TCAGTGGCCTGAACAGACAG-3′;

FBXO22-forward, 5′-GGAGCACCTTCGTGTTGAGT-3′;

FBXO22-reverse, 5′-ATCCAGGTTACGCTCCGATG-3′;

TRIM48-forward, 5′-CTGCTCTGTTTGCTGTGCTC-3′;

TRIM48-reverse, 5′-GTCTCCAAAAGCCTTCCAGTG-3′.

### 4.8. siRNA Knockdown

HEK293A cells were transfected with 10 nM non-targeting siRNA pool (Dharmacon, Lafayette, CO, USA) as control, siRNAs targeting either Skp1, Cullin1, FBXO22, or TRIM48 (GeneDesign, Osaka, Japan), and siRNA pools with a guaranteed knockdown efficiency of over 75% selected from Cherry-pick custom siRNA library targeting genes listed in Table S1 (Dharmacon) using Lipofectamine RNAiMAX Transfection Reagent (Invitrogen, Waltham, MA, USA), according to the manufacturer’s instructions.

Sequences of siRNAs used are as follows:

Skp1, 5′-GAGUUCUGAUGGAGAGAUAUU-3′;

Cullin1, 5′-GCCACUGAAUAAACAGGUA-3′;

FBXO22, 5′-GCACCUUCGUGUUGAGUAA-3′;

TRIM48, 5′-GAUAUUACUCUGCAUCACA-3.

### 4.9. Generation of Stable Cells

Stable cell lines that express 6Myc-TRIM48 or 6Myc-GPX4 were generated by retroviral transduction as described previously, with minor modifications [7]. Phoenix-AMPHO (the packaging cell line) was transfected with pMXs-IP [40] inserted with either 6Myc-TRIM48 [7], or 6Myc-GPX4 [41]. After 48 h, the growth medium that contained retrovirus was collected. HEK293 cells were incubated with the (virus-containing) medium with 10 µg/mL polybrene for 48 h, and uninfected cells were eliminated through puromycin selection.

### 4.10. Immunoprecipitation

The cells were lysed in IP lysis buffer. After centrifugation, their supernatants were immunoprecipitated with anti-Myc antibody (anti-Myc affinity M2 gel) (Wako). The immunoprecipitants were subsequently washed with IP lysis buffer, resuspended in laemmli buffer, and subjected to immunoblot analysis.

### 4.11. In Vivo Ubiquitination Assay

HEK293A cells transfected with the indicated plasmids were treated with 5 μM MG132 for 4 h before collection, lysed in IP lysis buffer supplemented with 10 mM N-ethylmaleimide (NEM), and subjected to immunoprecipitation with anti-Flag antibodies. The immunoprecipitates were washed with IP lysis buffer and then heated at 98 °C with lysis buffer containing 1% SDS to disrupt noncovalent protein–protein interactions. The heat-treated samples were subsequently diluted 10 times with IP lysis buffer, re-immunoprecipitated with anti-Flag antibodies (2nd-IP), and evaluated by immunoblot analysis.

### 4.12. Statistics

All data are presented as the mean ± standard deviation (SD). Statistical analysis was performed using GraphPad Prism software (version 9.5.1) (GraphPad, La Jolla, CA, USA). Two-group comparisons were analyzed by a two-tailed Student’s *t*-test, while multiple-group comparisons were analyzed by one-way or two-way ANOVA followed by Tukey’s or Dunnett’s test. Statistical significance was determined versus control unless otherwise noted. Values of * *p* < 0.05, ** *p* < 0.01, and *** *p* < 0.001 were considered statistically significant. NS, not significant.

## Data Availability

The original contributions presented in this study are included in the article/Supplementary Materials. Further inquiries can be directed to the corresponding author(s).

## Supplementary Materials

The following supporting information can be downloaded at: https://www.mdpi.com/article/10.3390/ijms26199472/s1.
