# Supplementary material for: FBXO22 Suppresses Oxidative Stress-Induced ASK1 Activation and Cell Death via Ubiquitination-Dependent Degradation of TRIM48
Source: Int J Mol Sci. 2025 Sep 27;26(19):9472. doi: 10.3390/ijms26199472 (PMC12524865; doi:10.3390/ijms26199472)
Supplement: Supplementary file 1 [file ijms-26-09472-s001.zip › ijms-3726791-supplementary.pdf]

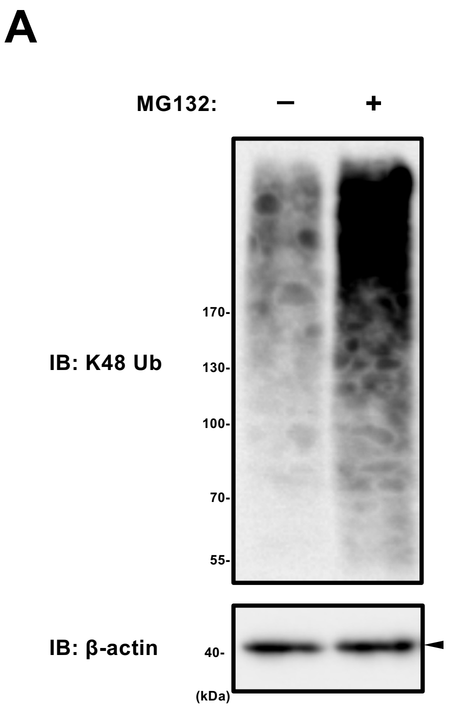

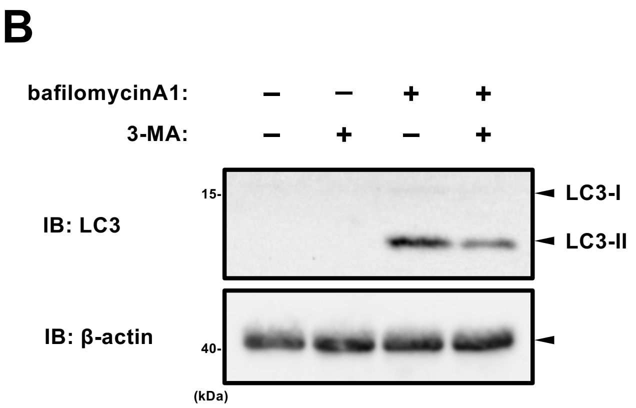

Supplementary Figure S1. Validation of proteasome and autophagy inhibition by MG132 and 3-MA.

(A) HEK293A cells stably expressing 6Myc-TRIM48 were treated with 5 μM MG132 for 4 hr. Cell lysates were immunoblotted with the indicated antibodies.

(B) HEK293A cells stably expressing 6Myc-TRIM48 were treated with 5 mM 3-MA and 10 nM bafilomycin A1 for 4 hr. Cell lysates were immunoblotted with the indicated antibodies.

Kashiwabara et al., Fig. S1.

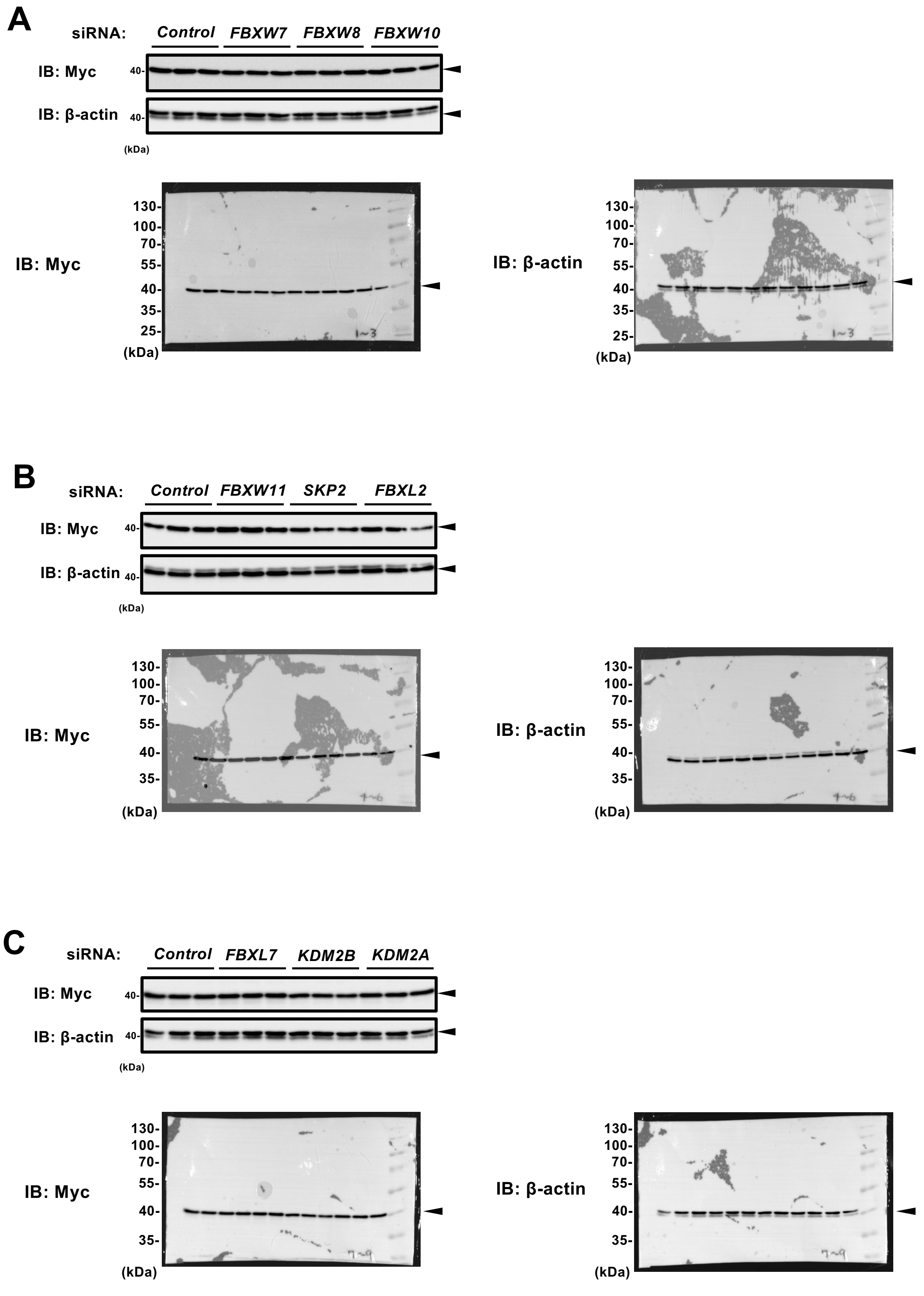

Kashiwabara et al., Fig. S2.

D

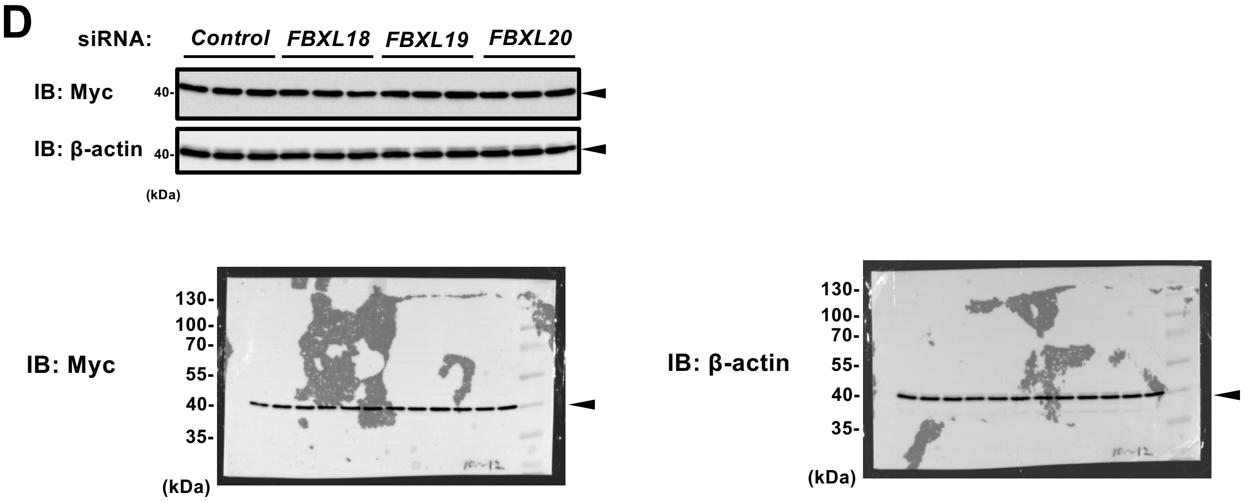

E

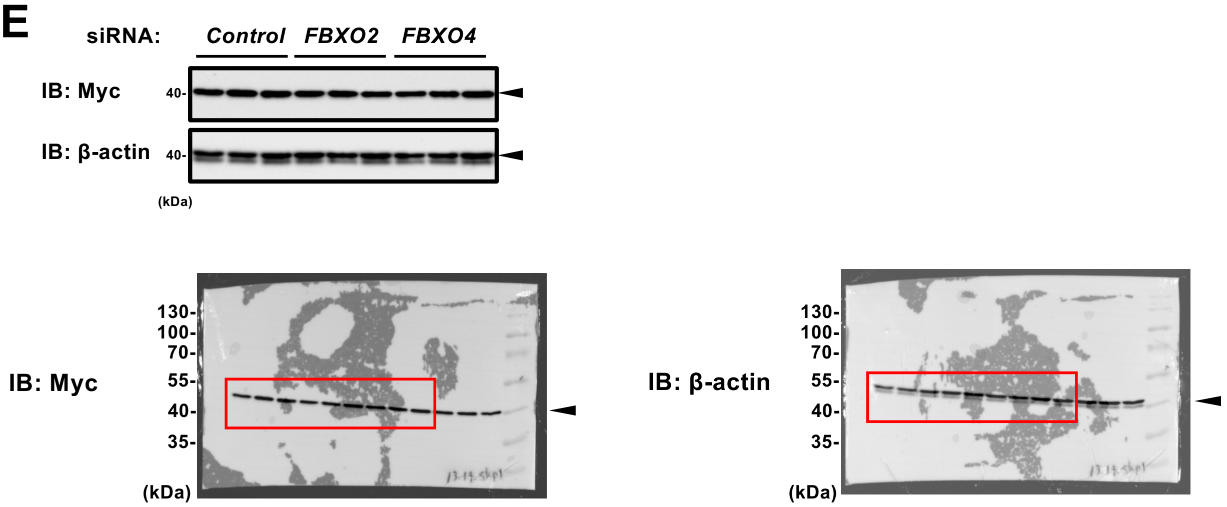

F

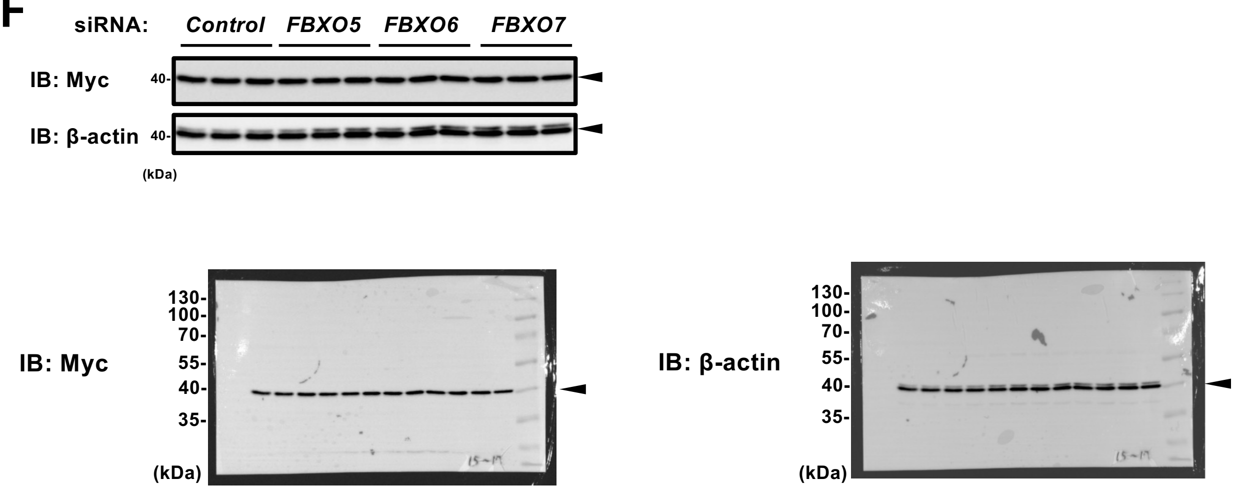

Kashiwabara et al., Fig. S2.

G

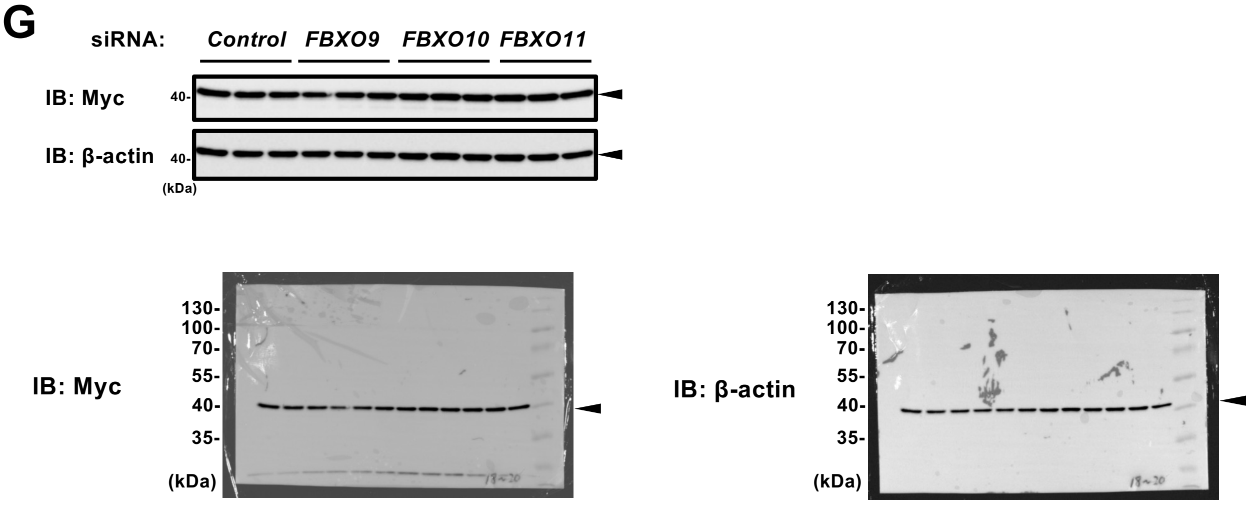

H

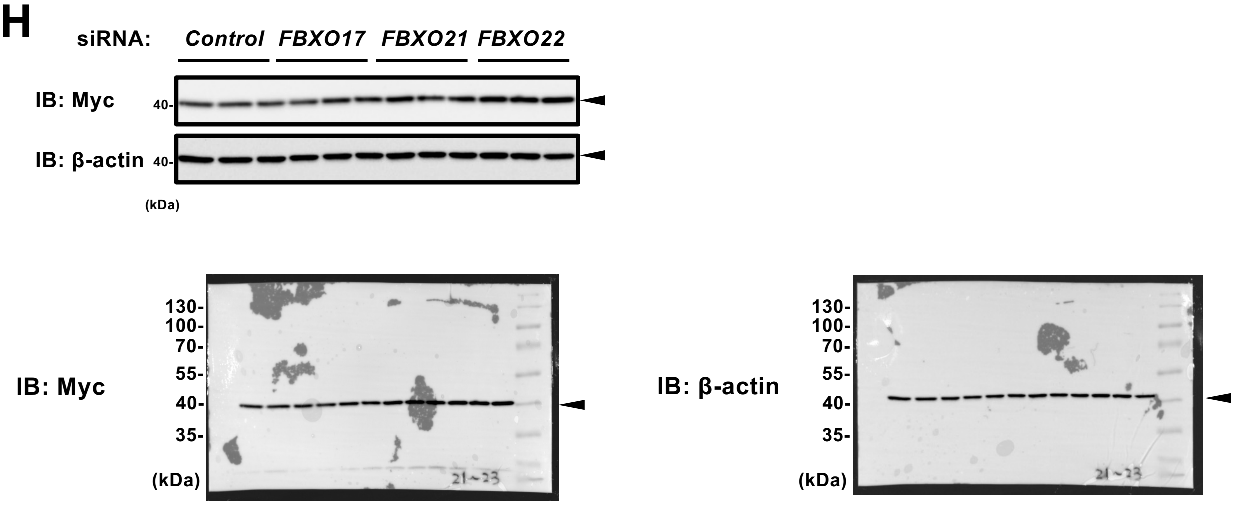

I

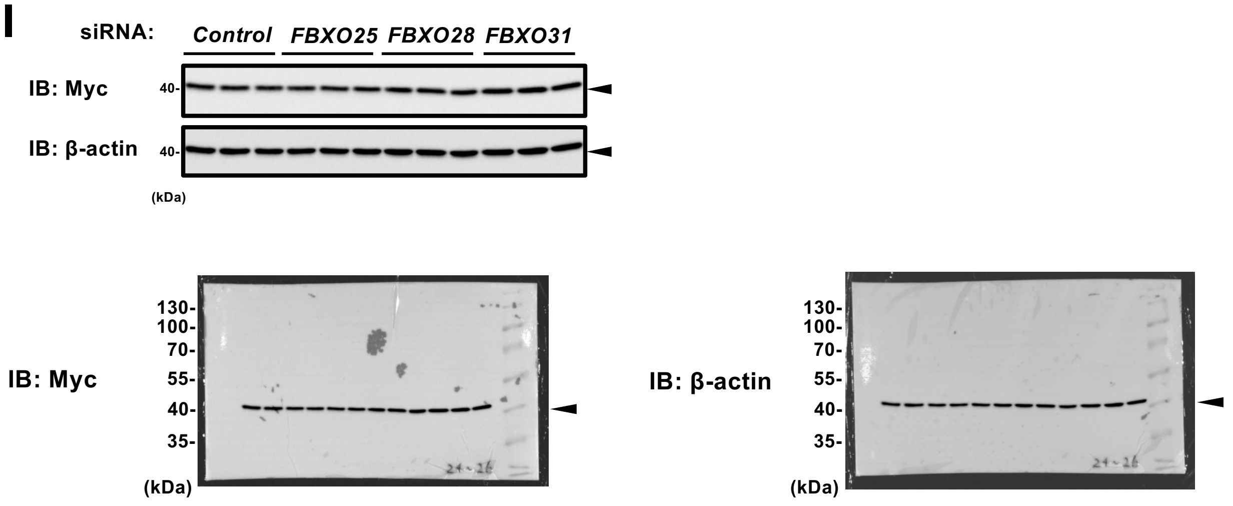

Kashiwabara et al., Fig. S2.

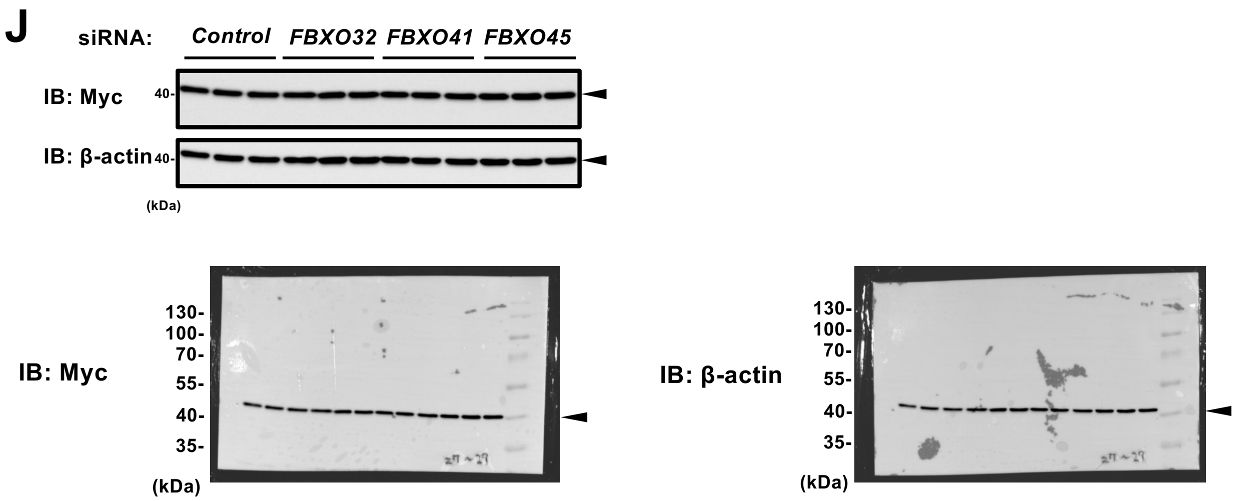

Supplementary Figure S2. An siRNA screen to identify F-box proteins targeting TRIM48 for degradation

(A-J) HEK293A cells stably expressing 6Myc-TRIM48 were transfected with 10 nM siRNAs targeting the indicated genes for 48 hr, and cell lysates were subjected to immunoblotting with the antibodies against Myc and β-actin.

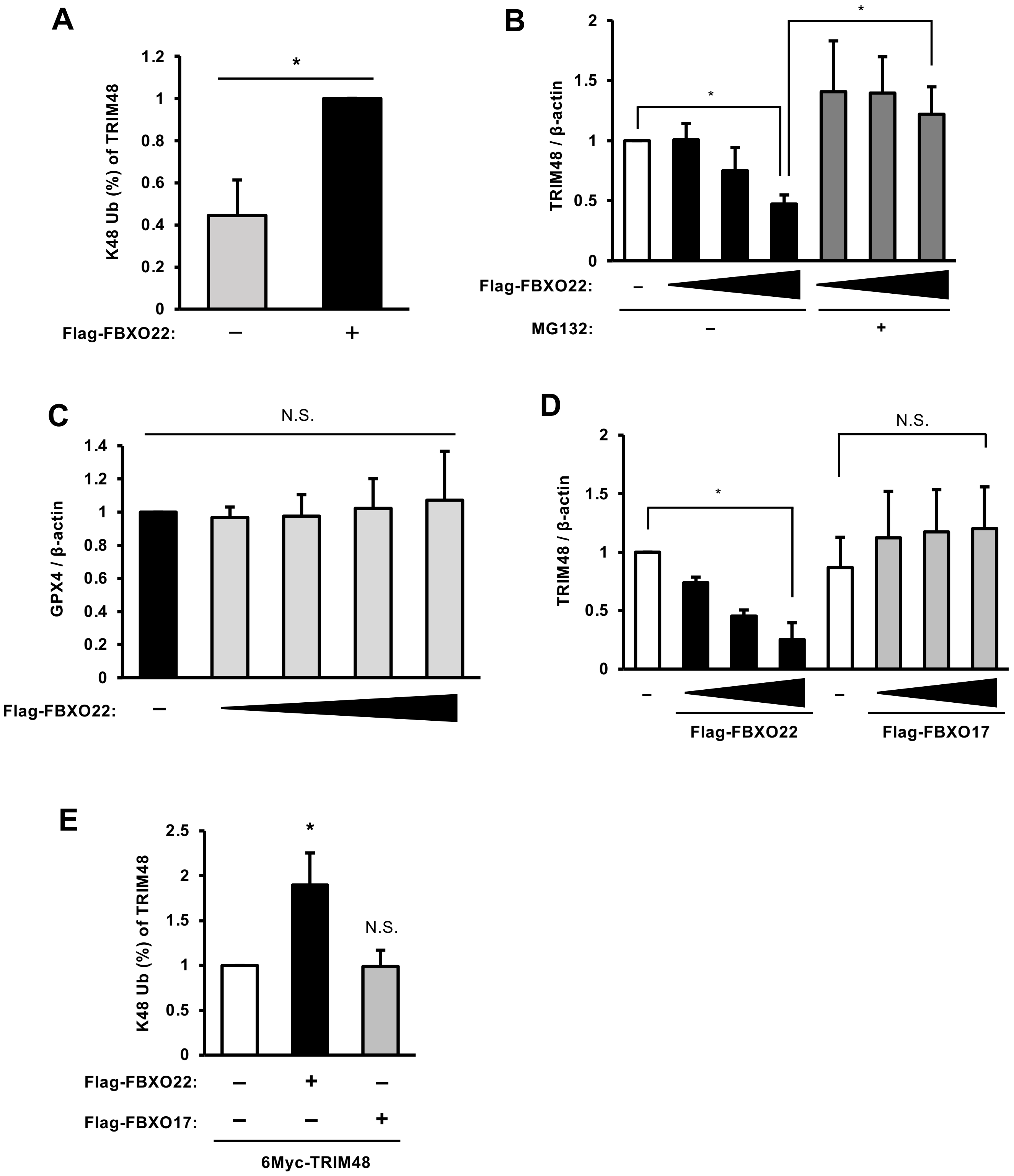

Supplementary Figure S3. Quantification of the immunoblot data

(A-E) Quantified immunoblot band intensities. Quantified band intensities of K48-linked Ub–TRIM48 were normalized against those of immunoprecipitated Myc-TRIM48 (A: Fig. 3A, E: Fig. 3F). (B-D) Quantified band intensities of Myc were normalized against those of β-actin (B: Fig. 3B, C: Fig. 3C, D: Fig. 3D). Data are shown as mean ± SD (n = 3).

Kashiwabara et al., Fig. S3.

A

__Fig. 1A__

IB: Myc

IB: β-actin

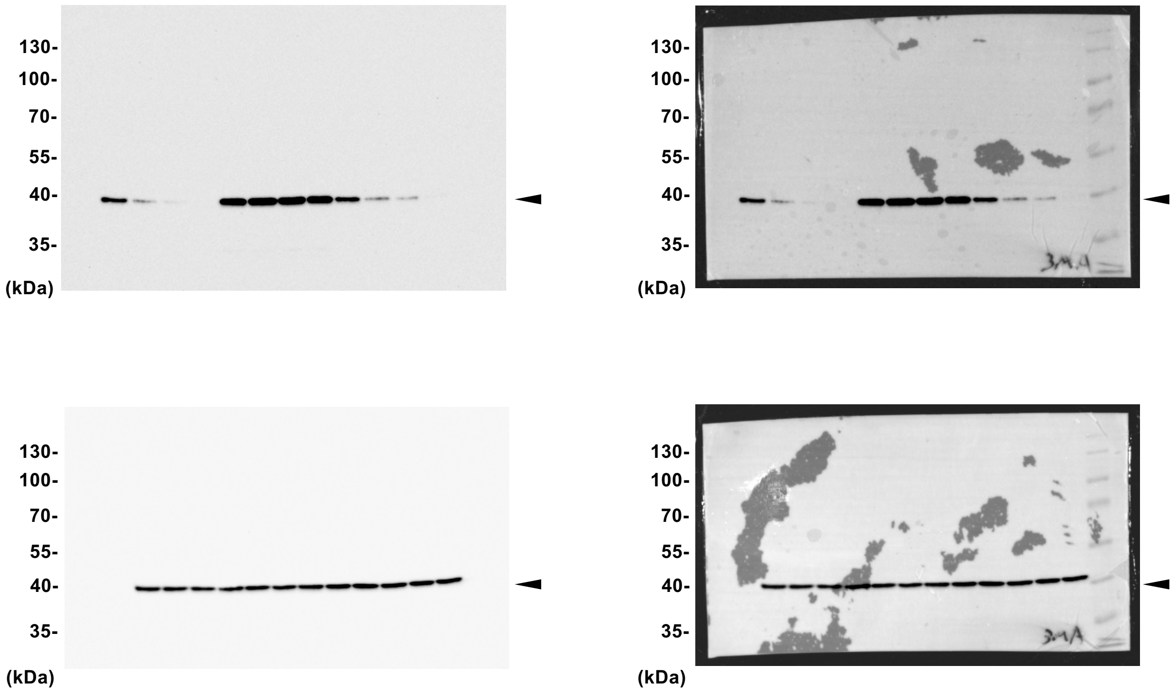

Kashiwabara et al., Fig. S4.

B

Fig. 1D

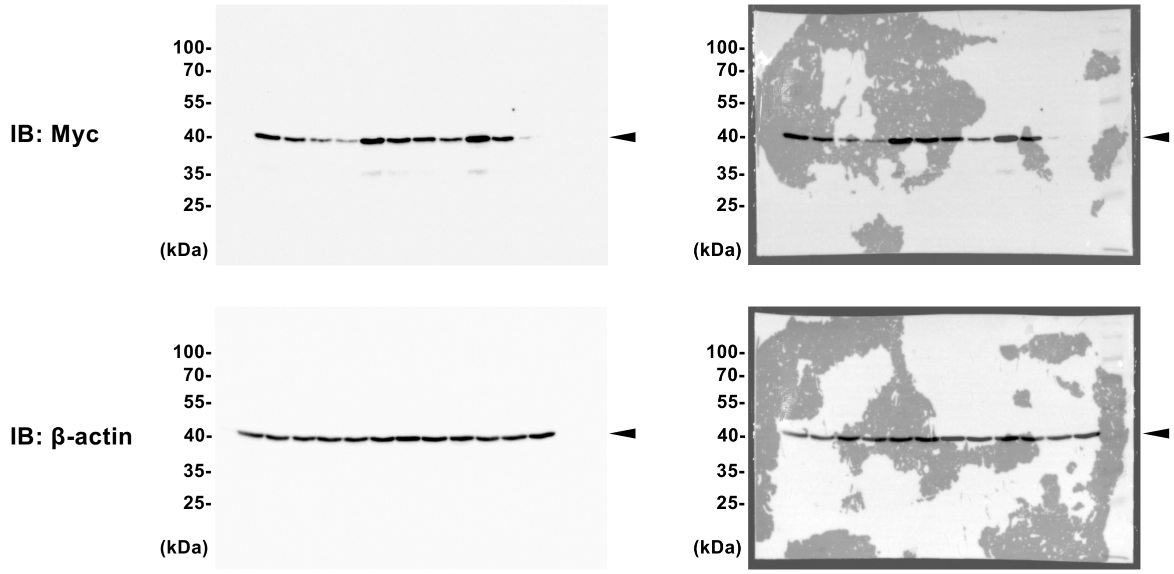

C

Fig. 1E

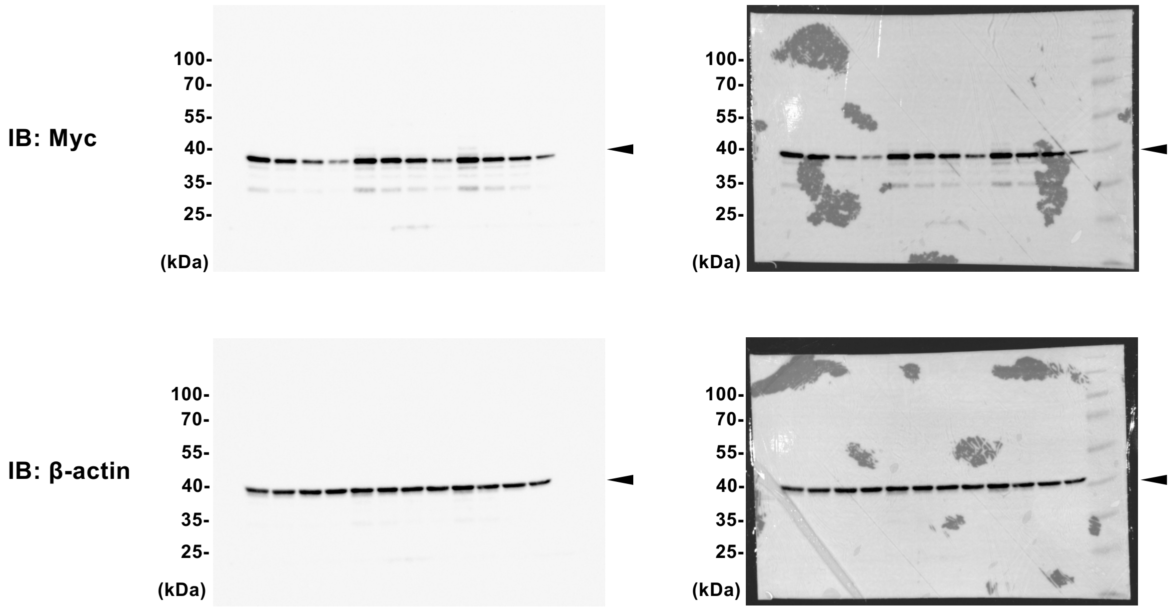

Kashiwabara et al., Fig. S4.

D

Fig. 2B

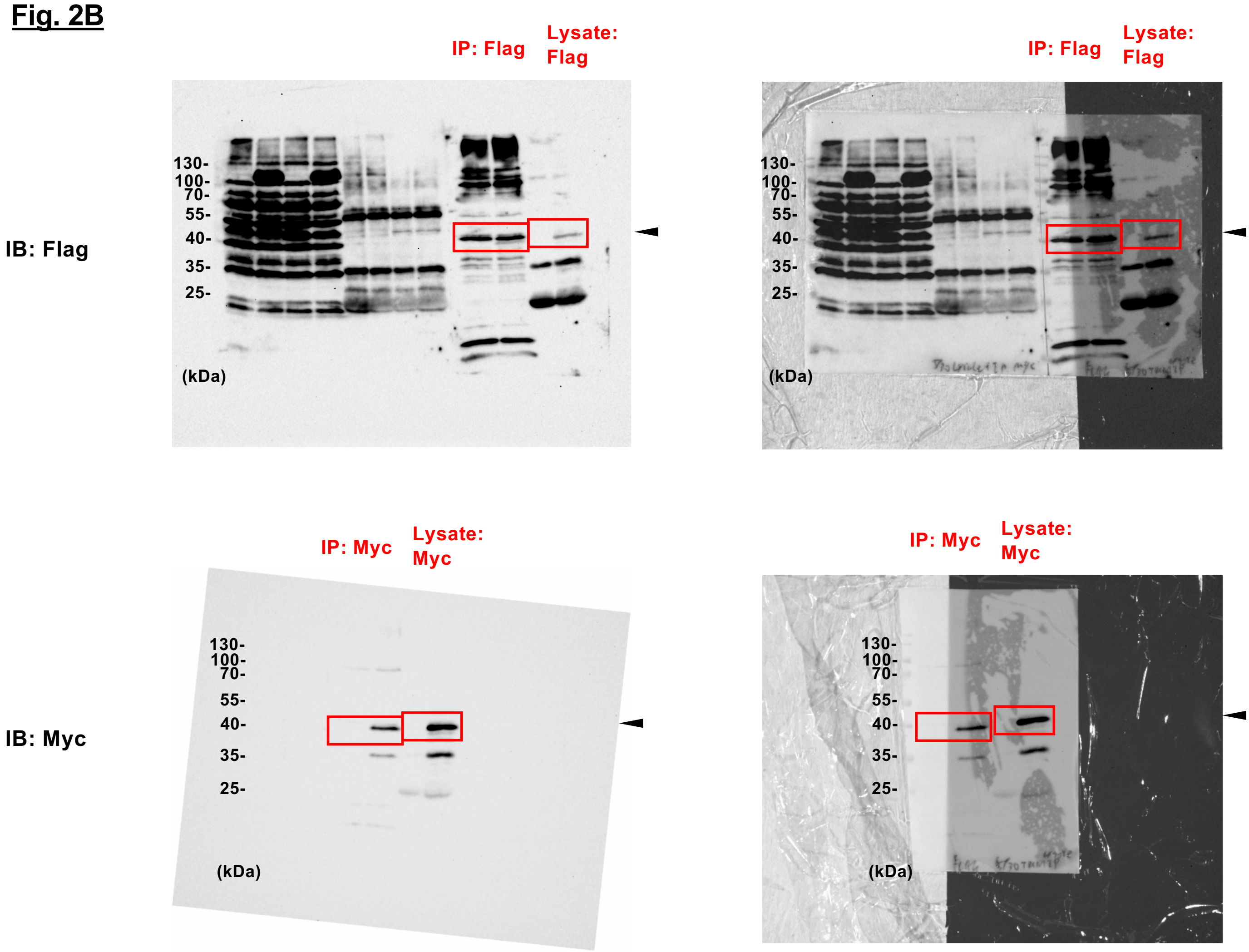

Kashiwabara et al., Fig. S4.

E

Fig. 3A

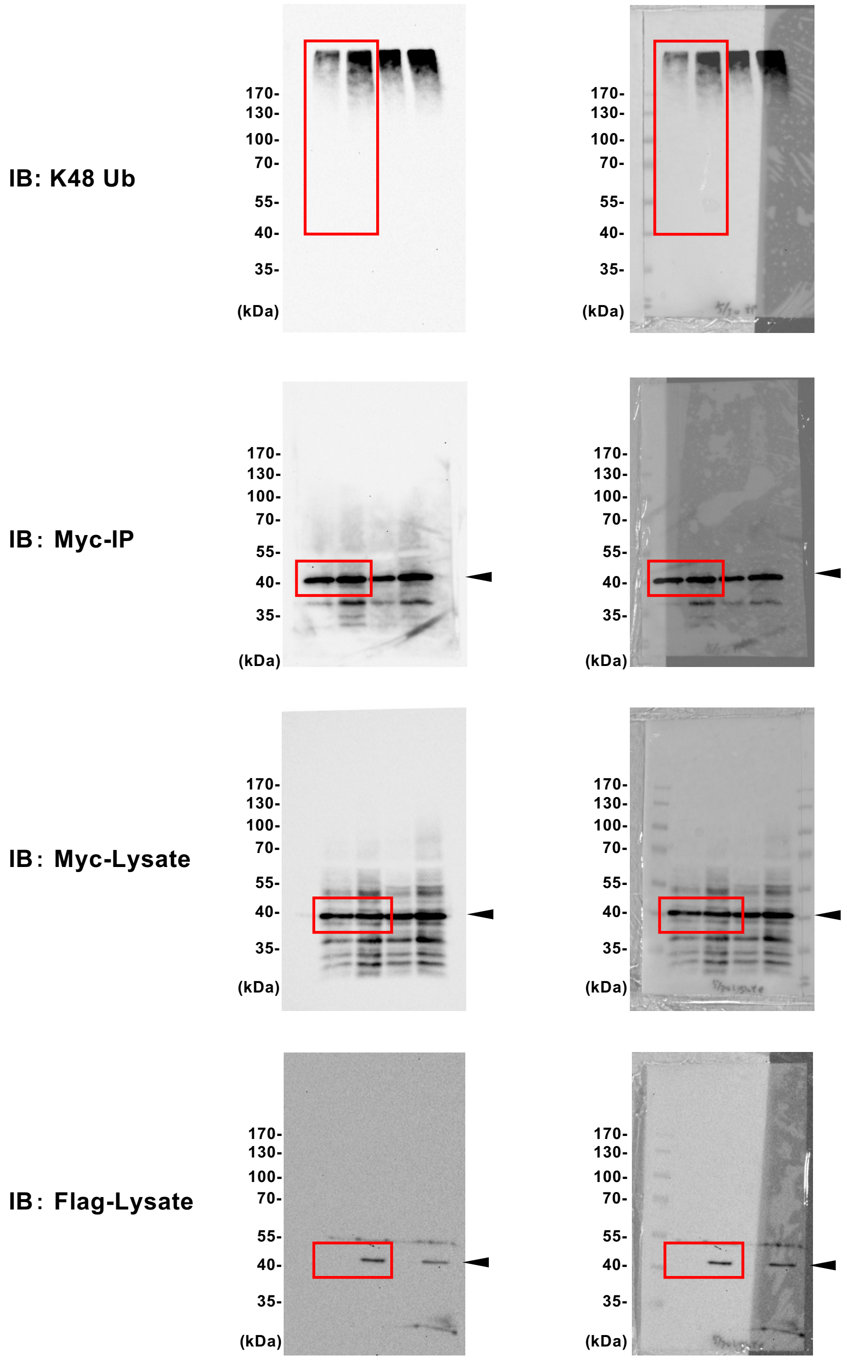

Kashiwabara et al., Fig. S4.

F

Fig. 3B

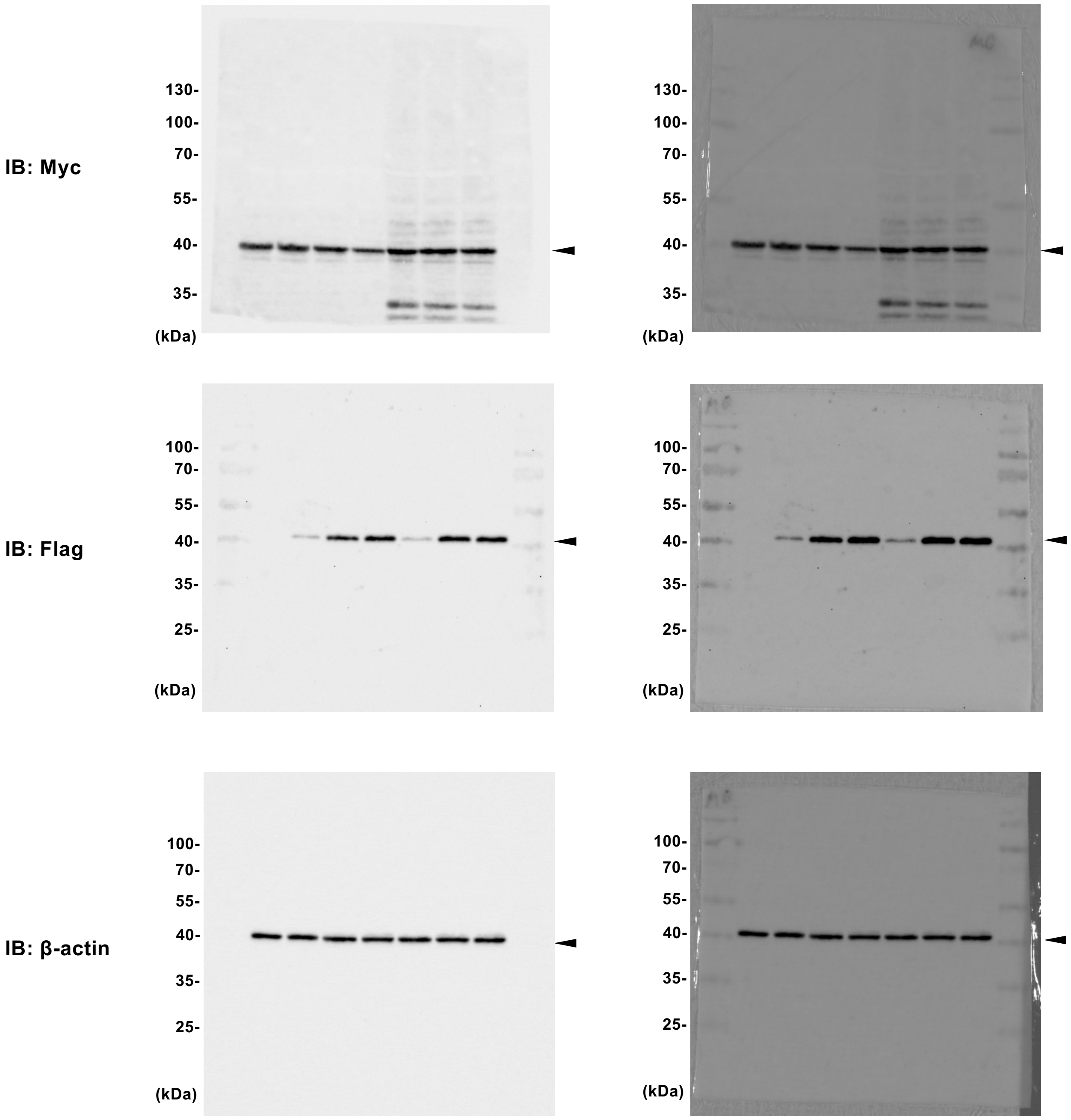

Kashiwabara et al., Fig. S4.

G

Fig. 3C

IB: Myc

IB: Flag

IB: β-actin

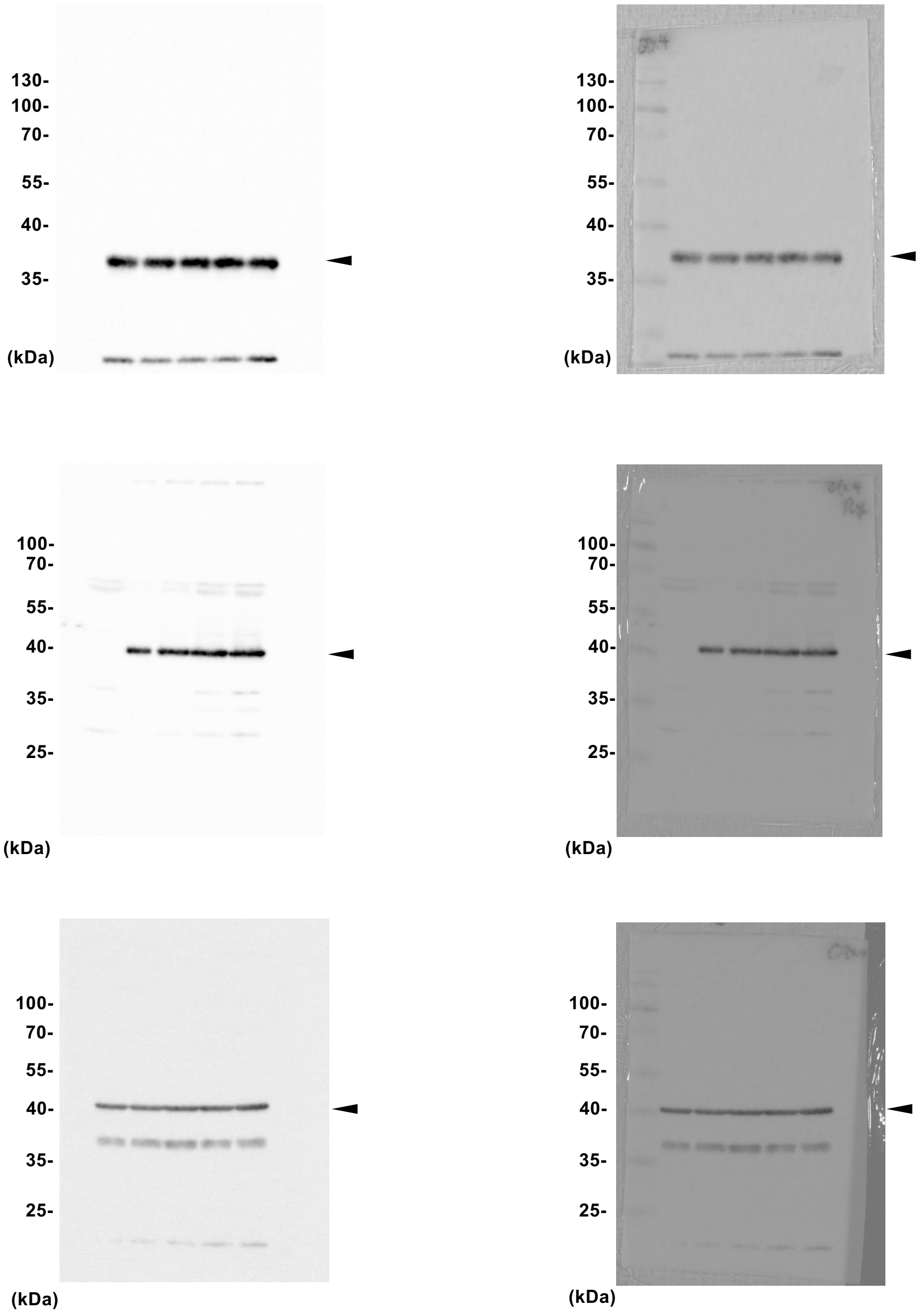

Kashiwabara et al., Fig. S4.

H

Fig. 3D

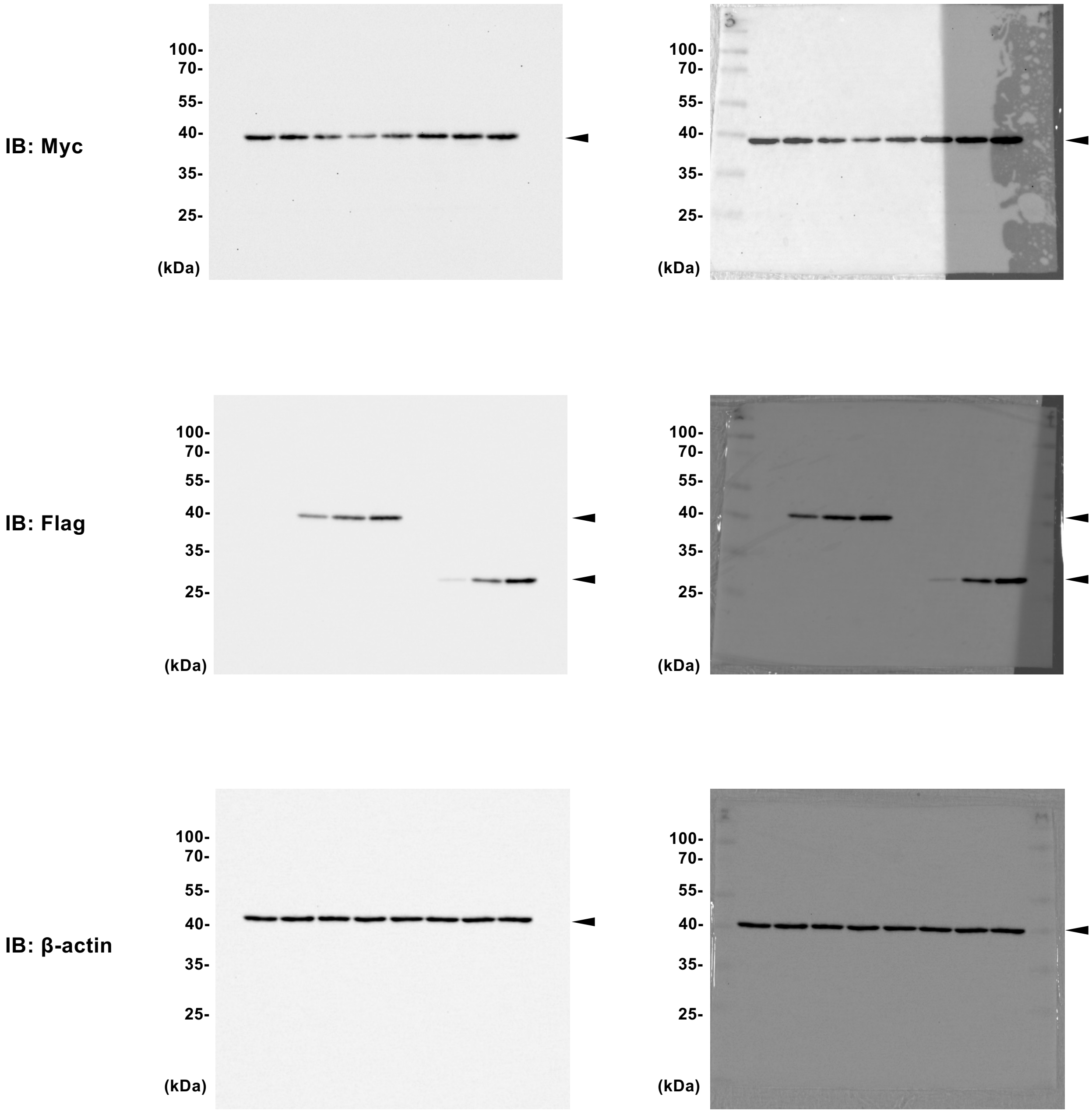

Kashiwabara et al., Fig. S4.

I

Fig. 3E

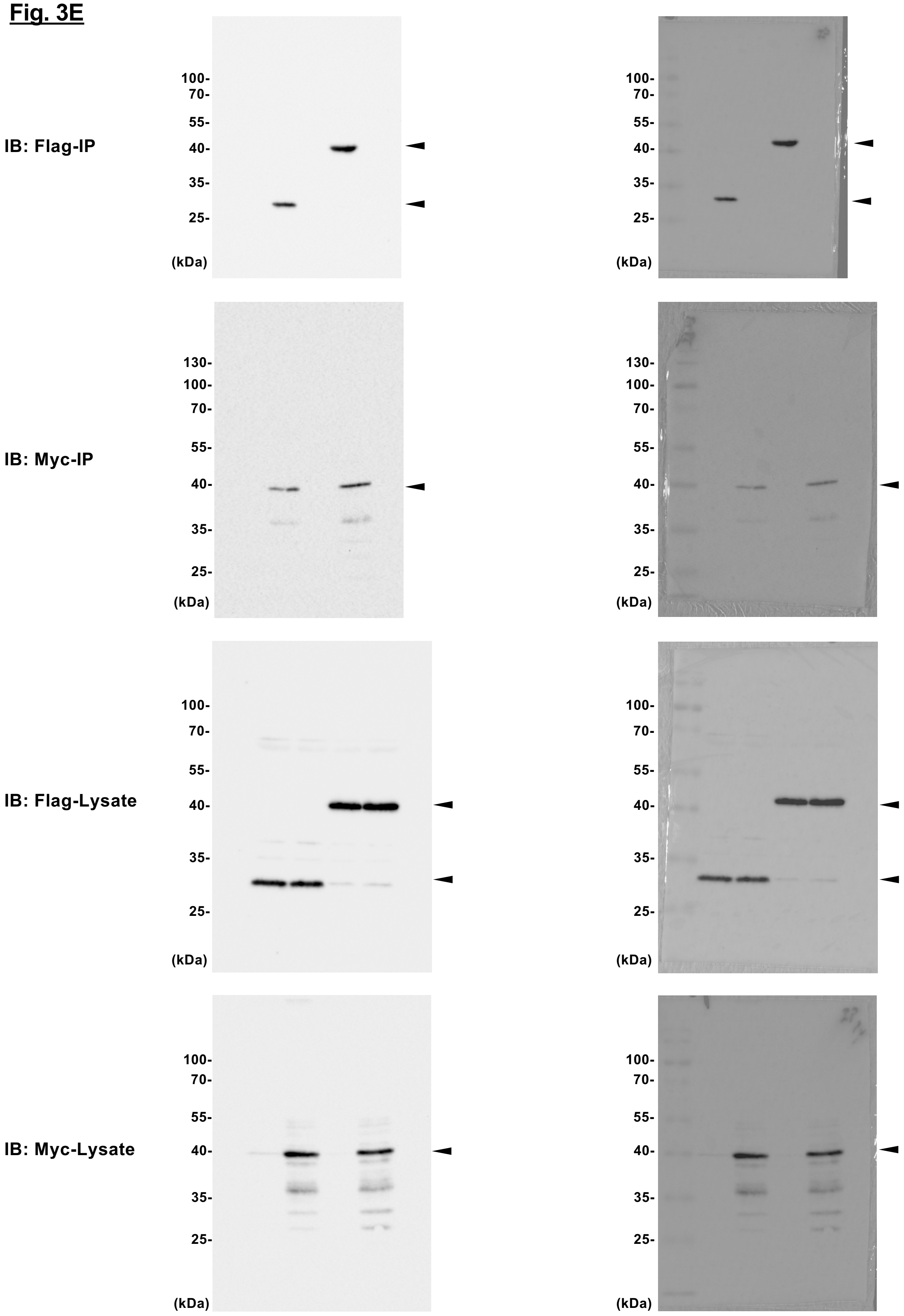

Kashiwabara et al., Fig. S4.

J

Fig. 3F

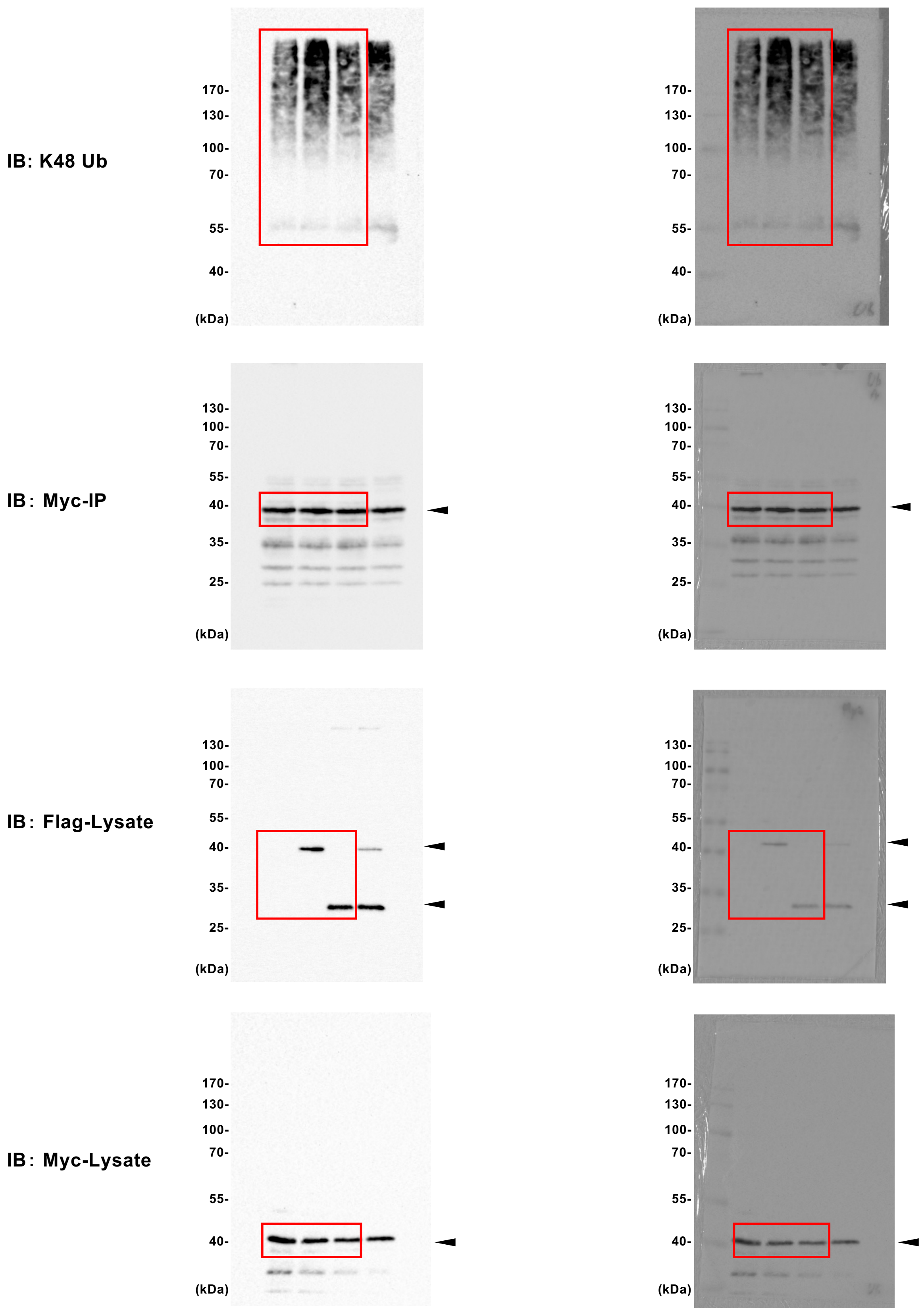

Kashiwabara et al., Fig. S4.

K

Fig. 4C

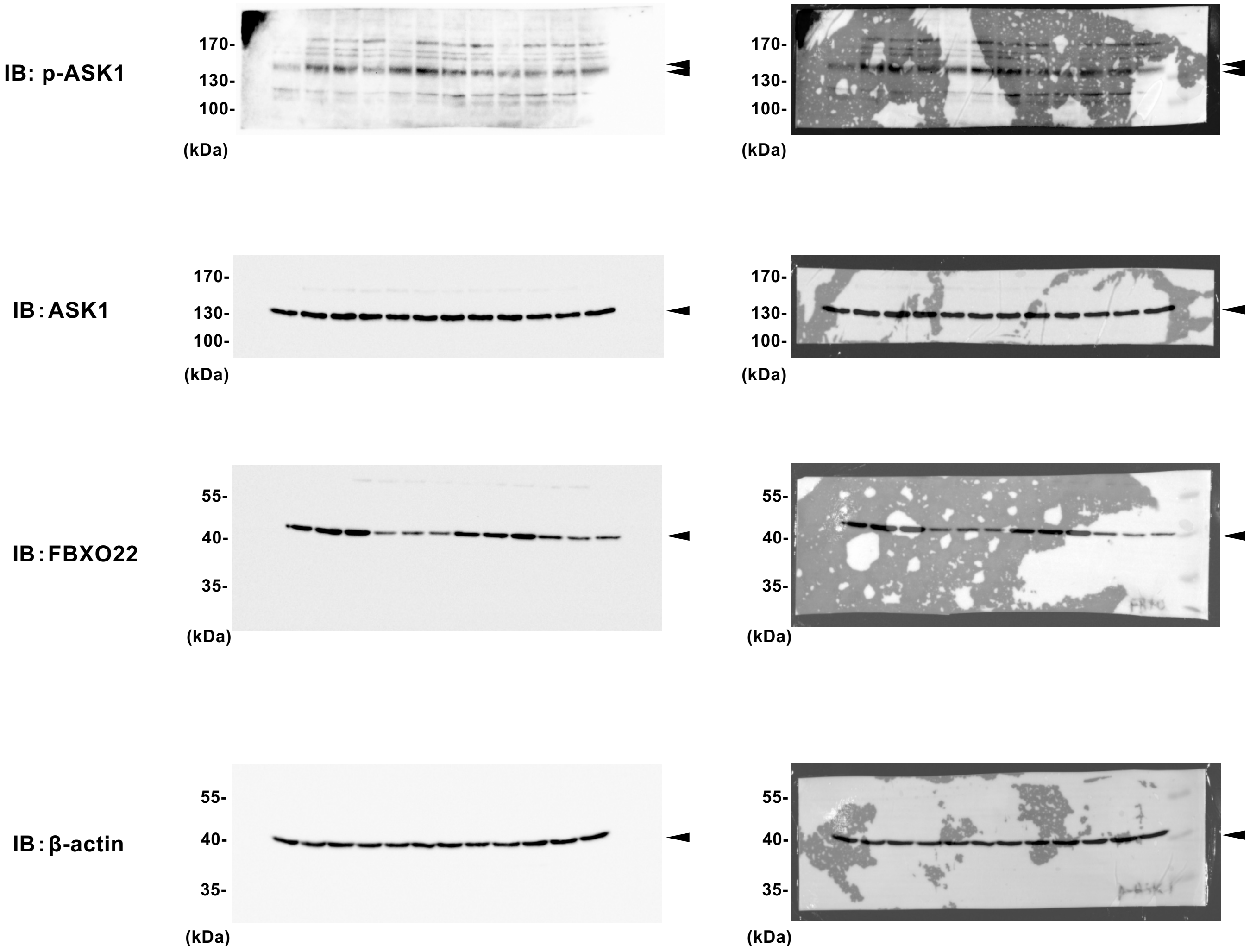

Kashiwabara et al., Fig. S4.

L

Fig. 4D

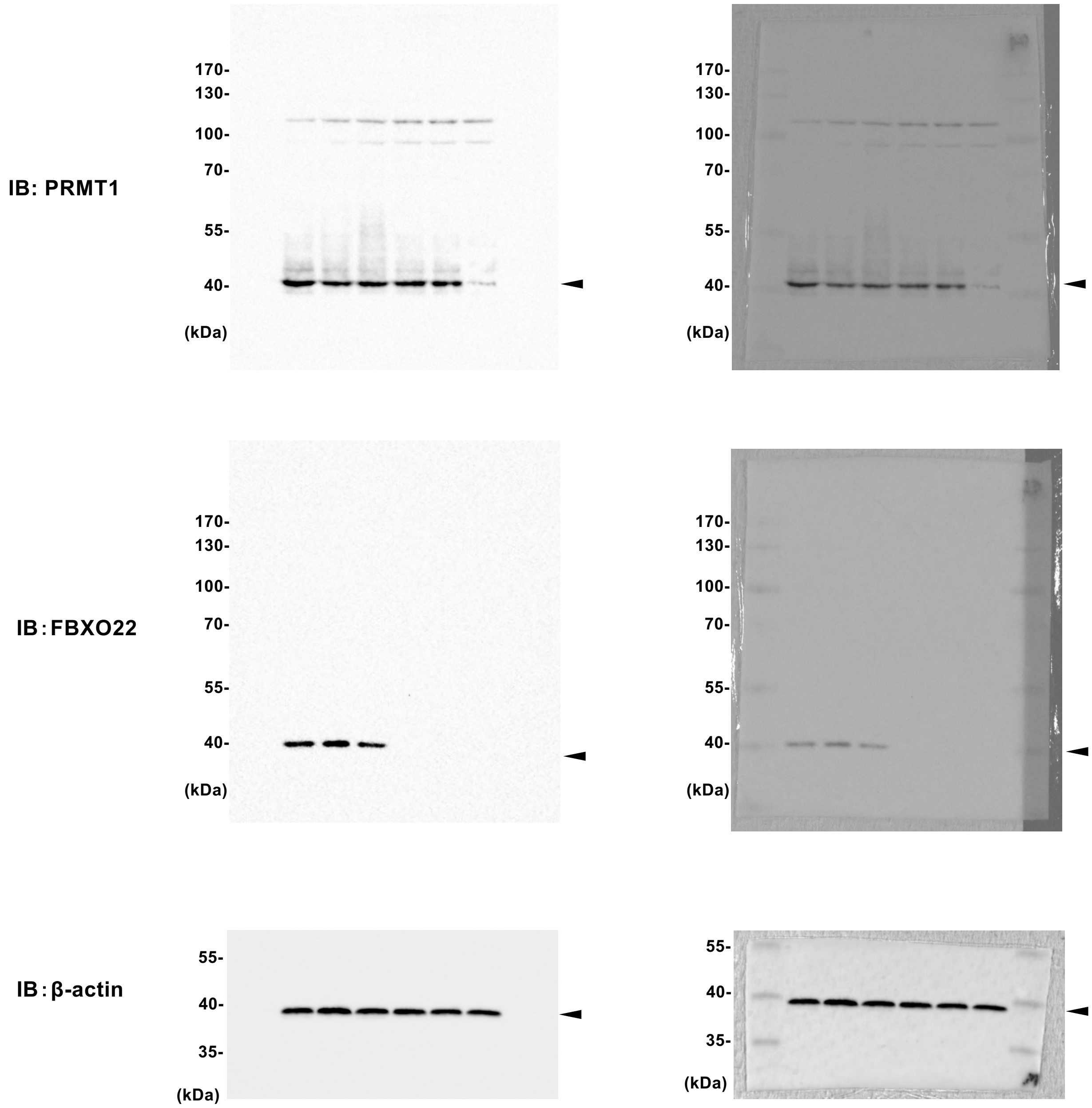

Kashiwabara et al., Fig. S4.

M

Fig. S1A

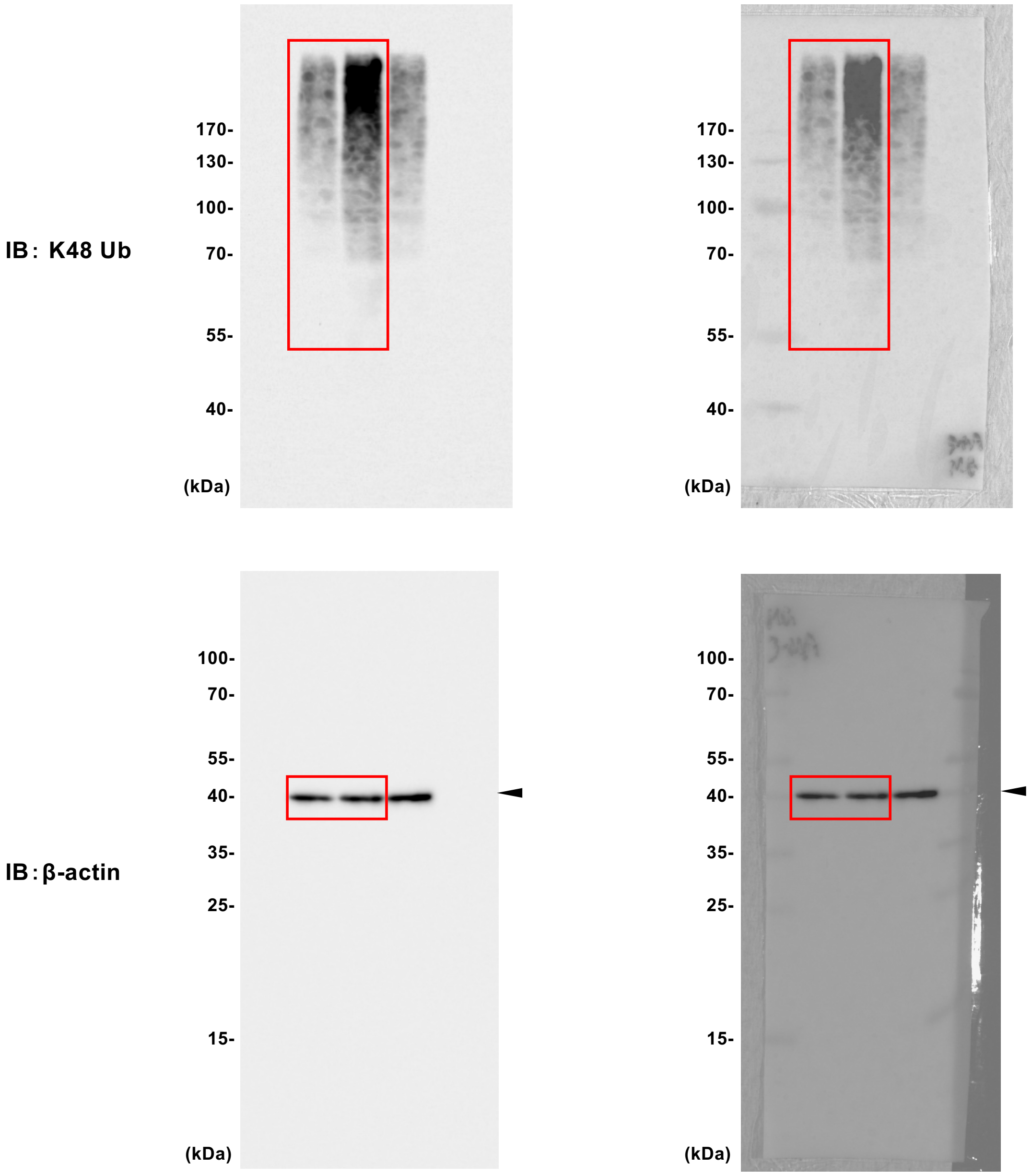

Kashiwabara et al., Fig. S4.

N

Fig. S1B

IB: LC3

IB: β-actin

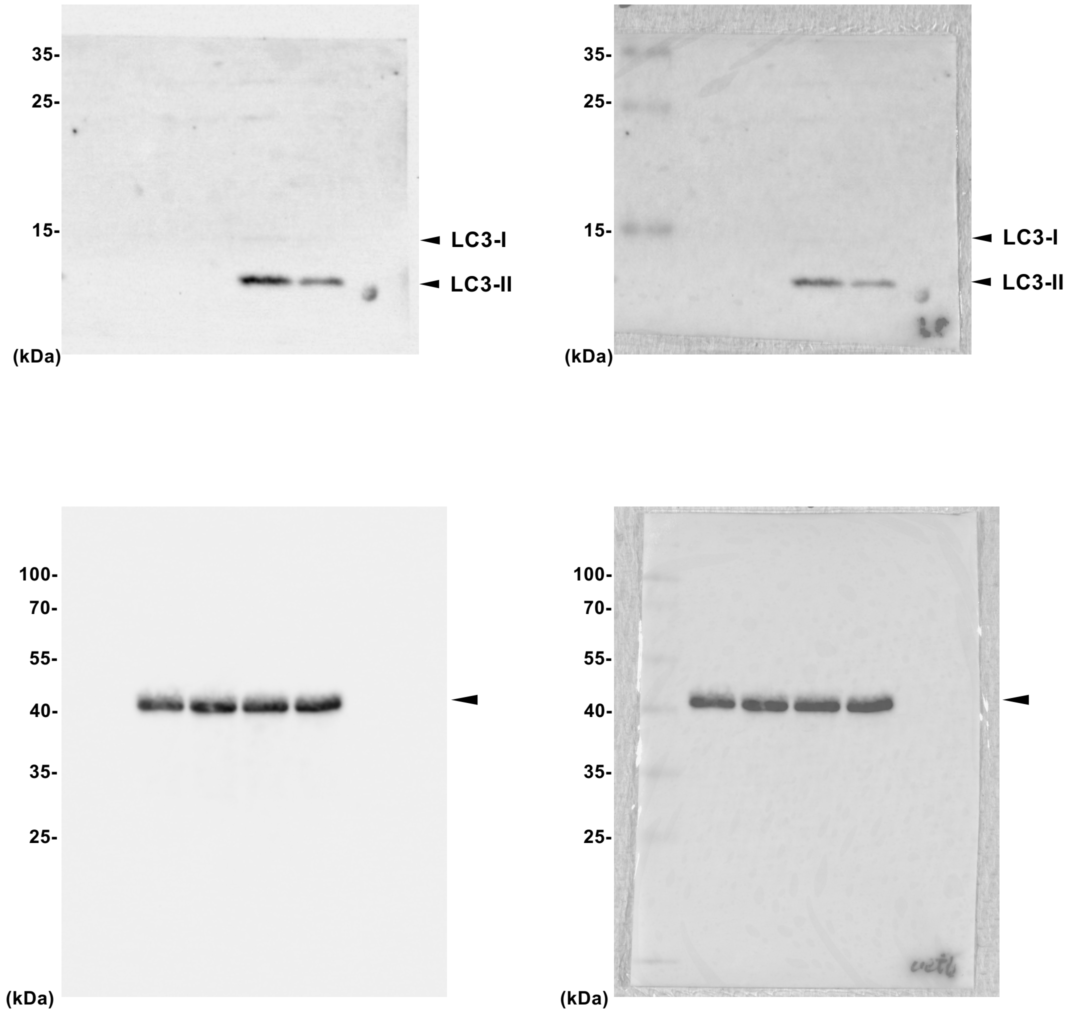

Supplementary Figure S4. Full scans of the immunoblot data

(A-N) Uncropped images of Fig. 1A (A), Fig. 1D (B), Fig. 1E (C), Fig. 2B (D), Fig. 3A (E), Fig.3B (F), Fig. 3C (G), Fig. 3D (H), Fig. 3E (I), Fig. 3F (J), Fig. 4C (K), Fig. 4D (L), Fig. S1A (M), and Fig. S1B (N).

Kashiwabara et al., Fig. S4.

Table S1. List of siRNAs used in Fig. 2A

Pool Catalog Number	Duplex Catalog Number	Gene Symbol	GENE ID	Gene Accession	GI Number	Sequence
L-004264-00	J-004264-07	FBXW7	55294	NM_001013415	61743925	CAACAACGACGCCGAAUUA
L-004264-00	J-004264-08	FBXW7	55294	NM_001013415	61743925	GGAGUUGUGUGGCGGAUCA
L-004264-00	J-004264-09	FBXW7	55294	NM_001013415	61743925	GUGAGUGGAUCUCUUGAUA
L-004264-00	J-004264-10	FBXW7	55294	NM_001013415	61743925	GGGCACCAGUCGUUAACAA
L-012431-00	J-012431-05	FBXW8	26259	NM_012174	30795120	GAGGAUGGGUUUCUUAAUA
L-012431-00	J-012431-06	FBXW8	26259	NM_012174	30795120	UGAGCACGAUGCAAGAAUA
L-012431-00	J-012431-07	FBXW8	26259	NM_012174	30795120	AGGAUGAGGUGCUGUGGUA
L-012431-00	J-012431-08	FBXW8	26259	NM_012174	30795120	CCAACUGCCUUACGAAUUG
L-014733-02	J-014733-21	FBXW10	10517	NM_001267586	389885063	AAAUGGUAGAUGACGGGAA
L-014733-02	J-014733-22	FBXW10	10517	NM_001267586	389885063	AGAAAGAACGGCCUCGCAU
L-014733-02	J-014733-23	FBXW10	10517	NM_001267586	389885063	CAUACAAGUGAAAGCGAUA
L-014733-02	J-014733-24	FBXW10	10517	NM_001267586	389885063	UGUAAGAACAGGCUCGUAU
L-003490-00	J-003490-05	FBXW11	23291	NM_033645	48928047	GCACAUUGGUGGAACAUUC
L-003490-00	J-003490-06	FBXW11	23291	NM_033645	48928047	GGACUUUAUUACCGCUUUA
L-003490-00	J-003490-07	FBXW11	23291	NM_033645	48928047	GAGCAAGGCUUAGAUCACA
L-003490-00	J-003490-08	FBXW11	23291	NM_033645	48928047	GAGCACCUGUGAAUUUGUU
L-003324-00	J-003324-14	SKP2	6502	NM_032637	16306593	UGUCAAUACUCUCGCAAAA
L-003324-00	J-003324-15	SKP2	6502	NM_032637	16306593	UCGGUGCUAUGAUAUAAUA
L-003324-00	J-003324-16	SKP2	6502	NM_032637	16306593	GGUAUCGCCUAGCGUCUGA
L-003324-00	J-003324-17	SKP2	6502	NM_032637	16306593	GGAUGUGACUGGUCGGUUG
L-013562-00	J-013562-05	FBXL2	25827	NM_012157	16306581	GCACAGAACUGCCGAAACA
L-013562-00	J-013562-06	FBXL2	25827	NM_012157	16306581	GCUCGGAAUUGCCACGAAU
L-013562-00	J-013562-07	FBXL2	25827	NM_012157	16306581	UCACUGACAGCACGUGUUA
L-013562-00	J-013562-08	FBXL2	25827	NM_012157	16306581	GAAGGUGUGGUGCAGAUAU
L-012457-00	J-012457-05	FBXL7	23194	NM_012304	21071079	GCAAACAGAUUUCCAUCCG
L-012457-00	J-012457-06	FBXL7	23194	NM_012304	21071079	UCAGGAUGCUCCAAAGUGA
L-012457-00	J-012457-07	FBXL7	23194	NM_012304	21071079	GUCUCAGGCUGUUACAAUA
L-012457-00	J-012457-08	FBXL7	23194	NM_012304	21071079	CAACCCGGCUUUCUUCUGA
L-014930-00	J-014930-05	KDM2B	84678	NM_001005366	54112379	GGGAGUCGAUGCUUAUUGA
L-014930-00	J-014930-06	KDM2B	84678	NM_001005366	54112379	CUCCAACGCUGCACAAUUU
L-014930-00	J-014930-07	KDM2B	84678	NM_001005366	54112379	CAACAGGCCAGGUCAGAUG
L-014930-00	J-014930-08	KDM2B	84678	NM_001005366	54112379	GCAAUAAGGUCACUGAUCA
L-012458-02	J-012458-17	KDM2A	22992	NM_001256405	373938466	GAACAAUCCCAGCGGCAAA
L-012458-02	J-012458-18	KDM2A	22992	NM_001256405	373938466	GUGUGCAAGACGUGGUAUA
L-012458-02	J-012458-19	KDM2A	22992	NM_001256405	373938466	CUGAGAAGAGAGACGCCAA
L-012458-02	J-012458-20	KDM2A	22992	NM_001256405	373938466	CAAAGAGCUCCACGGGACA
L-016587-00	J-016587-05	FBXL18	80028	NM_024963	21361980	GCUCCGGGCUGGUCAAUAU
L-016587-00	J-016587-06	FBXL18	80028	NM_024963	21361980	GAACACCUCGAGCUGAUUG
L-016587-00	J-016587-07	FBXL18	80028	NM_024963	21361980	GAACCUGCGGGUCUUCUAU
L-016587-00	J-016587-08	FBXL18	80028	NM_024963	21361980	GGACAUAUCCAAUGAUGAU
L-031874-00	J-031874-05	FBXL19	54620	NM_019085	42734336	GGGAGAAGCUAGAGCGUUU
L-031874-00	J-031874-06	FBXL19	54620	NM_019085	42734336	GAGAGAGGCACCUGAAGAA
L-031874-00	J-031874-07	FBXL19	54620	NM_019085	42734336	GGAUCGAGGAUGUUAAAGA
L-031874-00	J-031874-08	FBXL19	54620	NM_019085	42734336	GCACAUCGCUGAGUGAGGA
L-015029-00	J-015029-05	FBXL20	84961	NM_032875	14249619	GGCAAGAUGUUCUCAAUUA
L-015029-00	J-015029-06	FBXL20	84961	NM_032875	14249619	CGAAUUGACCUAUUUGAUU
L-015029-00	J-015029-07	FBXL20	84961	NM_032875	14249619	CAGAGGGUGCCAUAAGUUA
L-015029-00	J-015029-08	FBXL20	84961	NM_032875	14249619	GCAGGAACAUUGAAGUACU
L-012429-00	J-012429-05	FBXO2	26232	NM_012168	48995171	AUGAGAGCGUCAAGAAGUA
L-012429-00	J-012429-06	FBXO2	26232	NM_012168	48995171	CCGUUAAGCUACUGUCCGA
L-012429-00	J-012429-07	FBXO2	26232	NM_012168	48995171	GGAGCUGCCUGGAGACAGU
L-012429-00	J-012429-08	FBXO2	26232	NM_012168	48995171	GGUAGAACCCUGAGCGACC
L-012433-00	J-012433-05	FBXO4	26272	NM_012176	51093871	GAGCAUACAAGUGCAGUUA
L-012433-00	J-012433-06	FBXO4	26272	NM_012176	51093871	CGAUUGAUGUACAGCUAUA
L-012433-00	J-012433-07	FBXO4	26272	NM_012176	51093871	GCAGAUUGAUGGUAUUGGA
L-012433-00	J-012433-08	FBXO4	26272	NM_012176	51093871	GGAAGCCGGUACAGUGUGA
L-012434-00	J-012434-06	FBXO5	26271	NM_012177	15812190	CAACAGACACUUAAUAGUA
L-012434-00	J-012434-07	FBXO5	26271	NM_012177	15812190	CGAAGUGUCUCUGUAAUUA
L-012434-00	J-012434-08	FBXO5	26271	NM_012177	15812190	UGUAUUGGGUCACCGAUUG
L-012434-00	J-012434-09	FBXO5	26271	NM_012177	15812190	GAAUUUCGGUGACAGUCUA
L-013314-00	J-013314-05	FBXO6	26270	NM_018438	48995170	UGUCACAUCCUACGAAAUG
L-013314-00	J-013314-06	FBXO6	26270	NM_018438	48995170	GUACUGGGCAGGCUGGUAU
L-013314-00	J-013314-07	FBXO6	26270	NM_018438	48995170	GCACCUACCAACUCAAAGU
L-013314-00	J-013314-08	FBXO6	26270	NM_018438	48995170	GGACAGAGGUCUCCUACAC
L-013606-00	J-013606-05	FBXO7	25793	NM_001033024	74229028	GAAUGACGACAGUAUGUUA
L-013606-00	J-013606-06	FBXO7	25793	NM_001033024	74229028	CUGAGUCAAUUCAAGAUAA
L-013606-00	J-013606-07	FBXO7	25793	NM_001033024	74229028	CAUUAGAGACCUUGUAUCA
L-013606-00	J-013606-08	FBXO7	25793	NM_001033024	74229028	UAGCCAACAUAUACAAAGA
L-012469-00	J-012469-05	FBXO9	26268	NM_012347	53692182	CAAAUCAACUGGUGAGACU
L-012469-00	J-012469-06	FBXO9	26268	NM_012347	53692182	GUCCAUUGUUCCACGUUUA
L-012469-00	J-012469-07	FBXO9	26268	NM_012347	53692182	GCAACUUGUACCUGAUAUA
L-012469-00	J-012469-08	FBXO9	26268	NM_012347	53692182	UGUAAGCUCUAGCAAUUUA
L-026138-00	J-026138-05	FBXO10	26267	XM_291314	51467486	GAAAUACCAUCUACGCUAA
L-026138-00	J-026138-06	FBXO10	26267	XM_291314	51467486	CAACAGAGGCCACGGCAUU
L-026138-00	J-026138-07	FBXO10	26267	XM_291314	51467486	GCAGUGCGGUGUAUACAUA
L-026138-00	J-026138-08	FBXO10	26267	XM_291314	51467486	GAACUGGGCCUACAAGUAU
L-012428-00	J-012428-05	FBXO11	80204	NM_012167	30089921	GUAAAUUGUAGCCCUAUUA
L-012428-00	J-012428-06	FBXO11	80204	NM_012167	30089921	AAUAGUGACCCAACAAUAA
L-012428-00	J-012428-07	FBXO11	80204	NM_012167	30089921	GAAAGUUGCAAUAUACACA
L-012428-00	J-012428-08	FBXO11	80204	NM_012167	30089921	GCAAUGCAUUAGCAGGAAU
L-013128-00	J-013128-05	FBXO17	115290	NM_148169	22325387	GAACUGCGGCUGCGUCUAC
L-013128-00	J-013128-06	FBXO17	115290	NM_148169	22325387	GAACGGCUGGGCCAUAGAA
L-013128-00	J-013128-07	FBXO17	115290	NM_148169	22325387	CGACAGGUCUCCCACGUCU
L-013128-00	J-013128-08	FBXO17	115290	NM_148169	22325387	AAAAGGAAGUGGUCAAGUU
L-012917-00	J-012917-05	FBXO21	23014	NM_015002	53692181	GAACAUAGAUGAGUAAAGU
L-012917-00	J-012917-06	FBXO21	23014	NM_015002	53692181	UCAAUUGGUUGGAAGAGUA
L-012917-00	J-012917-07	FBXO21	23014	NM_015002	53692181	AGGAUGAACUGGUGUGUAU
L-012917-00	J-012917-08	FBXO21	23014	NM_015002	53692181	UCUUGAAGGUGCUGUAUAU
L-010812-00	J-010812-06	FBXO22	26263	NM_012170	22547147	GCACCUUCGUGUUGAGUAA
L-010812-00	J-010812-07	FBXO22	26263	NM_012170	22547147	GGUGGGAGCCAGUAAUUAU
L-010812-00	J-010812-08	FBXO22	26263	NM_012170	22547147	GUUCGCAUCUUACCACAUA
L-010812-00	J-010812-09	FBXO22	26263	NM_012170	22547147	GCUUAUGGAGGGAGUGUGU
L-019192-00	J-019192-05	FBXO25	26260	NM_012173	34878756	CGUUGUAUAUGCUUAGUGA
L-019192-00	J-019192-06	FBXO25	26260	NM_012173	34878756	UAUUUGGAUUUGCCGAUUA
L-019192-00	J-019192-07	FBXO25	26260	NM_012173	34878756	CCAGUUAACUUCAUUGAGU
L-019192-00	J-019192-08	FBXO25	26260	NM_012173	34878756	AAUCCUCGCUUAAUCAAAG
L-014080-00	J-014080-05	FBXO28	23219	NM_015176	7662157	GAAACAAGGCUGUCACUAU
L-014080-00	J-014080-06	FBXO28	23219	NM_015176	7662157	AAAGGUGAUUGAUGAGAUU
L-014080-00	J-014080-07	FBXO28	23219	NM_015176	7662157	GGAGAGGUACCAUAAUCUA
L-014080-00	J-014080-08	FBXO28	23219	NM_015176	7662157	UGAGAUAUGUCAAUUCUAC
L-016541-00	J-016541-05	FBXO31	79791	NM_024735	21362004	CCACGUCGAUGACCCUAUG
L-016541-00	J-016541-06	FBXO31	79791	NM_024735	21362004	UCACCGAUAUAGACACAUU
L-016541-00	J-016541-07	FBXO31	79791	NM_024735	21362004	GUGCAUGUACGGCCACAAA
L-016541-00	J-016541-08	FBXO31	79791	NM_024735	21362004	GGAGUAUGGUGUUUGCGAA
L-013005-00	J-013005-05	FBXO32	114907	NM_148177	22547143	GCAGAUCCGCAAACGAUUA
L-013005-00	J-013005-06	FBXO32	114907	NM_148177	22547143	GUACACUGGUCCAAAGAGU
L-013005-00	J-013005-07	FBXO32	114907	NM_148177	22547143	GUGCUGGUCGGGAACAUUA
L-013005-00	J-013005-08	FBXO32	114907	NM_148177	22547143	CAACUGAACAUCAUGCAGA
L-022720-00	J-022720-05	FBXO41	150726	XM_377742	51460632	GCUCCAAGUUCCUGGCAAU
L-022720-00	J-022720-06	FBXO41	150726	XM_377742	51460632	UCUCUAAGAUUCUGCACAU
L-022720-00	J-022720-07	FBXO41	150726	XM_377742	51460632	GGCCGUGCCUAACACAUAU
L-022720-00	J-022720-08	FBXO41	150726	XM_377742	51460632	GCAGAUGAUUGGUCGCUGU
L-023542-01	J-023542-09	FBXO45	200933	XM_946180	88971833	GGAGAAAGAAUUCGAGUCA
L-023542-01	J-023542-10	FBXO45	200933	XM_946180	88971833	ACACAUGGUUAUUGCGUAU
L-023542-01	J-023542-11	FBXO45	200933	XM_946180	88971833	AAACACAUGAAGUCGUAAA
L-023542-01	J-023542-12	FBXO45	200933	XM_946180	88971833	GUAUGAAACUGGUUGGGUU
